# A genetic atlas of relationships between circulating metabolites and liability to psychiatric conditions

**DOI:** 10.1038/s41380-026-03464-z

**Published:** 2026-02-06

**Authors:** Dylan J. Kiltschewskij, William R. Reay, Murray J. Cairns

**Affiliations:** 1 School of Biomedical Sciences and Pharmacy, The University of Newcastle, Callaghan, NSW Australia; 2 Precision Medicine Research Program, Hunter Medical Research Institute, New Lambton Heights, NSW Australia; 3 Menzies Institute for Medical Research, Hobart, TAS Australia

**Keywords:** Genetics, Psychiatric disorders, Biomarkers

## Abstract

Observational studies have reported alteration of circulating metabolites across several psychiatric conditions, but these studies cannot resolve causal relationships. Emerging evidence suggests a genetic relationship exists between these traits requiring further investigation to identify clinically actionable biology. Here, we used the largest genome-wide association studies available to investigate genetic correlation and causal relationships between 10 psychiatric conditions and 249 circulating metabolites. This revealed 1,100 significantly correlated trait pairings, involving fatty acids, lipoproteins and other metabolites, with evidence for causal effects on the liability for major depressive disorder, post-traumatic stress disorder and anorexia nervosa. Notably, the most robust association was a putative causal effect of high-density lipoprotein properties on anorexia nervosa. We also observed significant relationships between metabolic traits and cortical thickness and surface area, as well as evidence of shared gene-level common variant associations amongst 23 metabolite-psychiatric pairings, converging in pathways with metabolic and neuronal function. These findings highlight specific metabolites as potential biomarkers and therapeutic targets in the clinical management of psychiatric disorders.

## Introduction

Psychiatric conditions, such as schizophrenia, major depressive disorder and bipolar disorder, are associated with a variety of comorbid diagnoses that impede effective clinical management, increase mortality and decrease quality of life [1–3]. Individuals with a psychiatric condition exhibit strong overrepresentation of cardiometabolic conditions, including metabolic syndrome and coronary artery disease, which contribute to an 80% higher risk of premature death due to heart disease and a 10-to-20-year shorter life expectancy [1–3]. Alarmingly, while improved lifestyle and healthcare in recent decades have reduced the cardiometabolic burden in the general population, the incidence and mortality have remained stubbornly high amongst individuals with psychiatric illness [4, 5]. This is likely a reflection of the complex relationship between cardiometabolic traits and psychiatric illness, which is generally thought to arise from a combination of psychotropic medications, poor lifestyle, and systemic barriers to appropriate care [1–7]. Recent evidence suggests, however, that psychiatric and cardiometabolic traits share genetic components and causal relationships that make them targets for therapeutic intervention. This supports further investigation to capitalise on the potential therapeutic benefits for decreasing the burden of both the psychiatric symptoms and adverse cardiometabolic outcomes [8–14].

Large genome-wide association studies (GWAS) have uncovered strong common variant associations with psychiatric [15–24] and cardiometabolic traits [25, 26]. Recent advances in statistical genetics now provide the unprecedented opportunity to capitalise on the power of large GWAS to further explore shared biology in this comorbidity and prioritise interventions for clinical trials. For instance, GWAS-guided exploration of drug repurposing candidates suggests that drugs related to cardiometabolic health modify risk for some psychiatric conditions. Specifically, omega-3 nutraceuticals exhibit evidence for a beneficial effect in bipolar disorder, while antihypertensive angiotensin-converting enzyme (ACE) inhibitors may be risk increasing in schizophrenia [8]. We have also observed extensive genetic correlation amongst blood-based biomarkers, psychiatric illness and brain anatomy, with genetic causal inference further identifying causal relationships involving biomarkers such as glycaemic traits and C-reactive protein [9–11]. However, lipid-related traits – such as fatty acids, cholesterol, and phospholipids – and other metabolites remain relatively underexplored in this context, despite their intrinsic role in neuronal function and emergence as a key feature of psychiatric conditions [27, 28]. Indeed, many of these traits impact biological processes central to the pathogenesis of psychiatric illness, exhibit dysregulation in these conditions and, critically, can contribute to the development of cardiometabolic conditions [27, 28]. Although circulating metabolites represent an attractive prospect for preventative intervention, since many are clinically actionable through existing pharmacological and dietary interventions, the extent of shared genetic architecture and causal relationships between metabolites and psychiatric illness remains poorly characterised. To address this, we leveraged the largest uniformly processed GWAS of metabolites assayed via high-throughput metabolomics to comprehensively examine genetic correlation and causation between 249 circulating metabolites and 10 psychiatric conditions. Our analyses revealed extensive genetic correlation involving lipoproteins, phospholipids, triglycerides, fatty acids and other metabolic traits, while evidence for causal relationships was also uncovered. Furthermore, we report genes with common variant associations for both metabolites and psychiatric conditions that provide further insights into the interrelationship between these traits.

## Materials and methods

### GWAS summary statistics

**Fig. 1:**
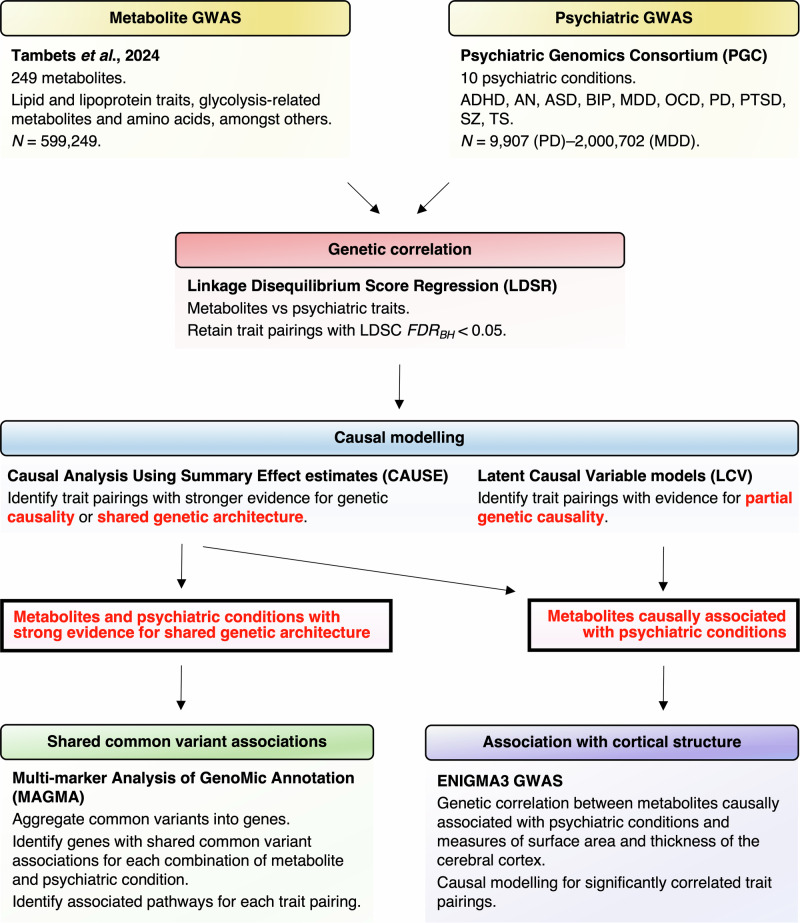
Schematic overview of study design. GWAS summary statistics for 249 circulating metabolites were obtained from a recent meta-analysis of ~600,000 individuals from the Estonian and UK Biobanks [25]. Summary statistics for 10 psychiatric conditions were obtained from the Psychiatric Genomics Consortium. Genetic correlation was firstly examined between all pairings of metabolite and psychiatric condition via linkage disequilibrium score regression (LDSR) [30, 31]. A total of 1100 significant trait pairings (*FDR*_*BH*_ < 0.05) were subjected to causal inference using Latent Causal Variable (LCV) [32] and Causal Analysis Using Summary Effect estimates (CAUSE) models [34]. Metabolites with evidence for causality were further examined for genetic relationships with respect to measures of cortical structure using GWAS from the ENIGMA consortium. CAUSE also identified 23 trait pairings with stronger evidence for shared genetic architecture than a causal relationship. Overlapping biology was further explored between these traits by identifying shared gene and gene-set associations via the Multi-marker Analysis of GenoMic Annotation (MAGMA) [35].

The aim of this work was to better understand how clinically actionable metabolites influence psychiatric health by examining their genetic relationship with psychiatric conditions using single nucleotide polymorphisms (SNPs; Fig. 1). For all metabolic and psychiatric traits analysed in this study, we selected GWAS datasets with the largest available sample size to maximise power for discovery across all methods described herein.

GWAS summary statistics containing SNP associations for 10 psychiatric conditions were obtained from the Psychiatric Genomics Consortium (PGC), prioritising the largest studies of predominantly European ancestry. We specifically examined attention deficit hyperactivity disorder (ADHD, *N* = 225,534 [15],), anorexia nervosa (AN, *N* = 72,517 [16],), autism spectrum disorders (ASD, *N* = 46,351 [17],), bipolar disorder (BIP, *N* = 840,309 [18],), major depressive disorder (MDD, *N* = 2,000,702 [19],), obsessive compulsive disorder (OCD, *N* = 9725 [20],), panic disorder (PD, *N* = 9907 [21],), post-traumatic stress disorder (PTSD, *N* = 1,249,840 [22],), schizophrenia (SZ, *N* = 130,644 [23],) and Tourette syndrome (TS, *N* = 14,307 [24],).

Metabolite GWAS summary statistics were sourced from a meta-analysis of 599,529 individuals from the Estonian and UK Biobanks covering 249 traits including lipids, amino acids and glycolysis-related metabolites [25]. For genetic causality, sensitivity analyses were conducted using an independent metabolite GWAS from [26], a meta-analysis of 136,016 individuals across 33 independent (non-Estonian or UK Biobank) cohorts. GWAS of surface area and thickness of the cerebral cortex (*N* = 33,992) were obtained from the ENIGMA consortium [29]. See Supplementary Methods for further details.

### Genetic correlation

Genetic correlation between all metabolites and psychiatric conditions was estimated via linkage disequilibrium score regression (LDSR) using the *ldsc* package (v1.0.1 [30, 31],). Summary statistics were firstly harmonised into a “munged” format, transforming SNP effect sizes and standard errors into *Z*-scores, and retaining approximately 1 million high-confidence HapMap3 SNPs outside the major histocompatibility complex region (MHC, chr6:28000000–34000000) with minor allele frequency (MAF) > 0.05. We then estimated the observed SNP heritability for each trait. For the primary analysis of metabolites and psychiatric conditions, all traits were significantly heritable (${h}_{{SNP}}^{2}$ *Z* > 5) and exceeded the recommended minimum for cross-trait LDSR (${h}_{{SNP}}^{2}$ *Z* > 4). Genetic correlation was subsequently estimated between traits using linkage disequilibrium (LD) scores from the 1000 Genomes Project European reference panel (available at: https://alkesgroup.broadinstitute.org/LDSCORE/). We note LDSR includes an intercept term that mitigates confounding due to factors such as sample overlap between GWAS. Trait pairings surpassing a Benjamini-Hochberg false discovery rate (*FDR*_*BH*_) < 0.05 were considered significant. See Supplementary Methods for further details.

### Latent causal variable modelling

We applied Latent Causal Variable (LCV) models to trait pairings with significant genetic correlation to assess partial genetic causality [32]. LCV estimates a posterior genetic causality proportion ($\overline{{GCP}}$), ranging from 0 (no causality) to ±1 (full causality), derived by comparing the mixed fourth moments (co-kurtosis) of SNP marginal effect size distributions between two traits. Positive $\overline{{GCP}}$ values suggest trait 1 is partially genetically causal for trait 2, while negative values suggest the opposite. As shown previously, trait pairings with |$\overline{{GCP}}$| ≥ 0.6, directionally consistent *Z*-scores and *FDR*_*BH*_ < 0.05 were considered to exhibit strong evidence for partial genetic causality [32]. Pairings with 0.5 ≤ |$\overline{{GCP}}$ | < 0.6 were deemed to have moderate evidence. For the primary analysis of metabolites on psychiatric conditions, heritability *Z* scores surpassed the recommended *Z* > 7 for all traits bar 16 (out of 232), which were marginally below this recommended cut-off (range = 5.9–7). We note that LCV also incorporates the cross-trait LDSR intercept term to reduce bias from sample overlap and non-genetic confounders. All summary statistics were munged prior to analysis as recommended [33]. See Supplementary Methods for further details.

### CAUSE

The Causal Analysis Using Summary Effect estimates (CAUSE) method (v1.2.0.0335 [34],) was also used to estimate causal relationships amongst genetically correlated traits. CAUSE is a Mendelian randomisation (MR) approach that uses SNPs associated with an exposure (i.e. metabolites) as instrumental variables to test evidence for causality with respect to an outcome (i.e. psychiatric conditions). It employs a Bayesian framework to model causal effects while accounting for confounding horizontal pleiotropy (i.e. SNPs acting directly on the outcome or via a confounding variable), and other confounders such as sample overlap, using a beta prior distribution.

Independent SNPs broadly associated with the exposure trait (*P*_GWAS_ < 1 ×10^–3^) were used to capture causal relationships and horizontal pleiotropy. We compared “causal” models (causality + pleiotropy) against “sharing” models (pleiotropy only) using the change in expected log pointwise posterior density (Δ*ELPD*) to assess model fit. In this context, negative Δ*ELPD* values indicate better fit for the causal model.

Three beta priors were tested, which assume high (α = 1, β = 2), moderate (α = 1, β = 10, default prior) or low correlated pleiotropy (α = 1, β = 50). Trait pairings were considered to show significant evidence for causality if Δ*ELPD* < 0 and *FDR*_*BH*_ < 0.05, with no evidence for reverse causality in models using the psychiatric trait as the exposure. Pairings with stronger evidence for shared genetic architecture were defined by Δ*ELPD* > 0 and *FDR*_*BH*_ < 0.05 in the forwards model, and *P* < 0.05 in the reverse model. See Supplementary Methods for further details.

### Gene and gene-set association analysis

We used the Multimarker Analysis of GenoMic Annotation (MAGMA, v1.10 [35],) to assess gene- and pathway-level associations shared by metabolite-psychiatric trait pairings with evidence of shared biology (as identified by CAUSE). GWAS SNPs were mapped to 19,240 autosomal protein-coding genes (NCBI GRCh37), extending gene boundaries by 5 kb upstream and 1.5 kb downstream to capture regulatory variants. Genes within the MHC region were excluded due to complex LD structure.

Gene-level associations were computed using MAGMA’s default test, which aggregates GWAS *P*-values into mean *χ*^2^ test-statistics for each gene. Gene associations with a Bonferroni-corrected *P* < 2.6 ×10^–6^ were deemed statistically significant, adjusting for 19,240 independent tests per phenotype. We then applied MAGMA’s competitive gene-set test to identify enriched canonical pathways from the Molecular Signatures Database (MSigDB, 3917 pathways), with *FDR*_*BH*_ < 0.05 deemed significant. Common significant genes and pathways were compared between each pairing of metabolite and psychiatric trait to explore shared biology.

Shared genetic architecture was further examined via pairwise meta-analysis of genic *Z*-scores for each metabolite-psychiatric trait pairing using Stouffer’s weighted *Z* method. To account for sample overlap and other confounders, the LDSR intercept for each trait pairing was included as a covariate. Gene-set meta-analyses were additionally conducted using the same model. See Supplementary Methods for further details.

## Results

### Extensive genetic correlation between circulating metabolites and psychiatric conditions

Genetic correlation was examined between 249 blood-based metabolites and 10 psychiatric conditions using LDSR, leveraging the largest uniformly processed metabolite GWAS to date [25]. The metabolites include 81 lipid, amino acid, and glycolysis-related traits (e.g. total triglycerides, tyrosine and glucose), herein referred to as “primary metabolites” (See Table S1 for a full overview of all traits, including heritability estimates). The remaining traits relate to lipoprotein absolute lipid content (98 traits) or lipid ratios (70 traits), subcategorised by lipoprotein diameter (e.g. triglycerides in small, medium, large and very large high density lipoprotein (HDL) [25]).

**Fig. 2:**
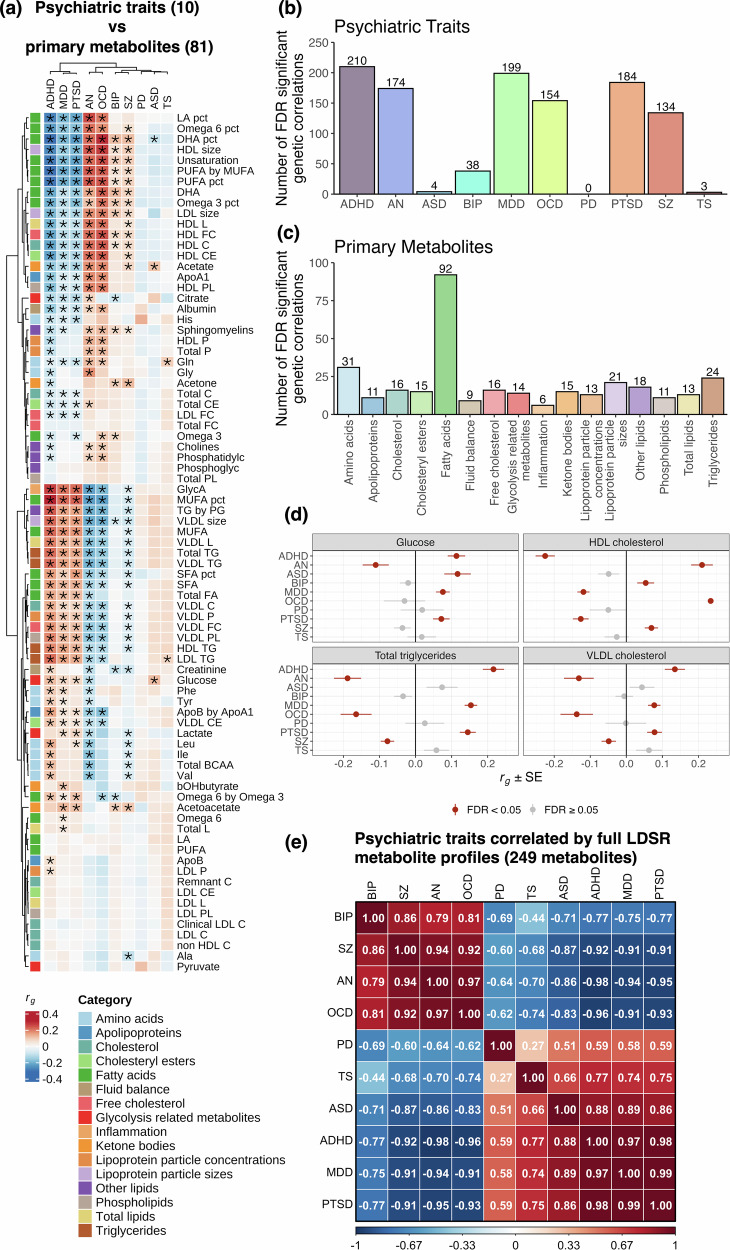
Genetic correlation amongst circulating metabolites and psychiatric traits. (**a**) Heatmap depicting LDSR genetic correlation coefficients (*r*_*g*_) between the 81 primary metabolites and 10 psychiatric conditions. Rows and columns were subject to hierarchical clustering to identify similar groups of traits. *FDR_*BH*_ < 0.05. Full metabolite names can be accessed in Table S1, and data for the lipoprotein traits subcategorised by particle diameter can be accessed in Table S2 and Fig. S1. (**b**) The number of significant metabolite correlations (including lipoprotein traits subcategorised by particle diameter) per psychiatric trait. (**c**) The number of significant psychiatric correlations for the 81 primary metabolites. (**d**) Forest plots depicting key examples of metabolic traits exhibiting divergent patterns of genetic correlation with respect to psychiatric traits. Data plotted as *r*_*g*_ ± standard error (*SE*). (**e**) Heatmap of Pearson correlation amongst psychiatric traits upon pairwise comparison of metabolite LDSR genetic correlation profiles (transformed into *Z*-scores [i.e. *r*_*g*_ / *SE*]). Rows and columns were subject to hierarchical clustering to identify psychiatric conditions with the most similar metabolite correlation profiles.

A total of 1100 metabolite-psychiatric trait pairings were significantly correlated (*FDR*_*BH*_ < 0.05; Fig. 2a, Fig S1, Table S2). Interestingly, all psychiatric conditions except PD were correlated with at least one metabolite, with particularly strong representation for ADHD (210 metabolites), AN (174), MDD (199), OCD (154), PTSD (184) and SZ (134; Fig. 2b). Among the primary metabolites, fatty acid traits were most frequently correlated with psychiatric illness (92 correlations), followed by amino acids (31), triglycerides (24) and lipoprotein particle sizes (21; Fig. 2c, Table S2). We also observed extensive correlation of lipoprotein absolute lipid content (430 correlations) and lipid ratios (345) across a range of lipoprotein subclasses (Fig. S1, Table S2). However, none of these metabolite categories were significantly enriched or underrepresented for correlations with any given psychiatric condition after correction for multiple testing (Table S3). The top three genetic correlations ranked by absolute *Z* scores are as follows: glycoprotein acetyls and MDD (*r*_*g*_ = 0.2, *SE* = 0.02, *P* = 2.11 ×10^–38^), ratio of monounsaturated fatty acids to total fatty acids and MDD (*r*_*g*_ = 0.22, *SE* = 0.02, *P* = 1.2 ×10^–34^) and degree of unsaturation and ADHD (*r*_*g*_ = –0.3, *SE* = 0.02, *P* = 1.66 ×10^–35^; Table S2).

Many metabolites exhibited divergent patterns of genetic correlation across conditions (Fig. 2d). For instance, total triglycerides and very low-density lipoprotein (VLDL) cholesterol were positively correlated with ADHD, MDD and PTSD, and negatively correlated with AN, OCD and SZ (Fig. 2d). Hierarchical clustering of LDSR profiles (transformed into *Z*-scores. i.e. *r*_*g*_ / *SE*) revealed two major groups of psychiatric conditions with opposing metabolite correlation patterns (Fig. 2e, Table S4). Specifically, AN, BIP, OCD and SZ were positively correlated with each other (Pearson *r* ≥ 0.79, *P* ≤ 2.36 ×10^–55^), and negatively correlated with the remaining six psychiatric conditions (*r* ≤ –0.44, *P* ≤ 1.71 ×10^–13^). Among these six conditions, ADHD, MDD and PTSD exhibited particularly strong correlation (*r* ≥ 0.97, *P* ≤ 1.02 ×10^–157^). These findings suggest widespread and condition-specific genetic correlations between circulating metabolites and psychiatric traits.

### Evidence for metabolites exhibiting partial genetic causality on psychiatric conditions

We assessed partial genetic causality between significantly correlated metabolites and psychiatric conditions using LCV models [32]. Causality was quantified using the posterior genetic causality proportion ($\overline{{GCP}}$), which reflects the strength, not magnitude, of genetic causation.

Two trait pairings exhibited strong evidence for partial genetic causality ($\overline{{|GCP|}}$ ≥ 0.6, *FDR*_*BH*_ < 0.05): phospholipids to total lipids ratio in chylomicrons and extremely large VLDL → MDD ($\overline{{GCP}}$ = 0.65) and cholesterol to total lipids ratio in medium low-density lipoprotein (LDL) → PTSD ($\overline{{GCP}}$ = 0.63; Table 1, Table S5). By integrating the corresponding LDSR genetic correlation coefficients, we can infer that elevation of these metabolites is respectively associated with increased risk for MDD (*r*_*g*_ = 0.16) and decreased risk for PTSD (*r*_*g*_ = –0.19; Table 1, Table S2). A sensitivity analysis using independent metabolite GWAS [26] revealed directionally consistent $\overline{{GCP}}$ estimates for these relationships ( ≥ 0.18; Table S6). While this did not exceed criteria for strong evidence of partial genetic causality, we note these GWAS are of substantially reduced sample size (*N* = 136,016) compared to those used for the primary analysis (*N* = 599,529).

**Table 1:** Metabolites and psychiatric conditions with strong or moderate evidence of partial genetic causality.

Metabolite	Psychiatric condition	*r*_*g*_^a^	$\overline{{GCP}}$^b^	*SE*^c^	*Z*^d^	*P*^e^	*FDR*_*BH*_^f^
Phospholipids to total lipids ratio in chylomicrons and extremely large VLDL	MDD	0.16	0.649	0.172	11.578	4.12 × 10^−20^	3.37 × 10^−17^
Cholesterol to total lipids ratio in medium LDL	PTSD	−0.188	0.635	0.198	5.396	4.66 × 10^−7^	9.55 × 10^−5^
Cholesterol to total lipids ratio in large VLDL	PTSD	−0.138	0.583	0.233	4.976	2.74 × 10^−6^	2.8 × 10^−4^
Free cholesterol to total lipids ratio in large VLDL	OCD	−0.194	0.581	0.204	3.661	4.05 × 10^−4^	0.022
Cholesterol in small VLDL	OCD	−0.134	0.575	0.21	5.036	2.14 × 10^−6^	2.59 × 10^−4^
Free cholesterol to total lipids ratio in small HDL	ADHD	−0.197	0.558	0.182	5.297	7.11 × 10^−7^	1.17 × 10^−4^
Docosahexaenoic acid	ADHD	−0.212	0.558	0.134	10.792	2.06 × 10^−18^	8.43 × 10^−16^
Free cholesterol in medium HDL	AN	0.176	0.534	0.207	3.863	2 × 10^−4^	0.012
Free cholesterol to total lipids ratio in very large VLDL	PTSD	−0.18	0.532	0.201	4.644	1.05 × 10^−5^	9.59 × 10^−4^
Cholesterol to total lipids ratio in large LDL	ADHD	−0.292	0.529	0.159	4.357	3.22 × 10^−5^	0.003
Ratio of omega-3 fatty acids to total fatty acids	ADHD	−0.207	0.528	0.129	8.333	4.63 × 10^−13^	1.26 × 10^−10^
Total lipids in HDL	AN	0.198	0.524	0.199	4.147	7.11 × 10^−5^	0.005

^a^The genetic correlation coefficient estimated via LDSR. Since all $\overline{{GCP}}$ values in this table are > 0 (suggesting the metabolite is the putative causal factor), r_g_ denotes whether higher metabolite levels are associated with increased or decreased risk of the corresponding psychiatric condition.

^b^The posterior genetic causality proportion estimate. $\overline{{GCP}}$ values > 0 (with directionally consistent Z-scores and FDR_BH_ < 0.05) suggest partial genetic causality of the metabolite on the corresponding psychiatric condition. $\overline{{GCP}}$ > 0.6 and 0.5 ≤ $\overline{{GCP}}$ < 0.6 were deemed strong and moderate evidence, respectively.

^c^Standard error of the posterior genetic causality proportion estimate.

^d^Z-score of the posterior genetic causality proportion ($\overline{{GCP}}$ / SE).

^e^P-value testing whether the $\overline{{GCP}}$ is significantly different from zero.

^f^The Benjamini-Hochberg false discovery rate.

A further 10 metabolites exhibited moderate evidence for partial genetic causality (0.5 ≤ $\overline{{GCP}}$ < 0.6; Table 1, Table S5). Four were associated with ADHD: docosahexaenoic acid, free cholesterol to total lipids ratio in small HDL, cholesterol to total lipids ratio in large LDL, and ratio of omega-3 fatty acids to total fatty acids. The remaining relationships included two HDL-related traits → AN, two VLDL-related traits → OCD and a further two VLDL-related traits → PTSD (Table 1, Table S5). Most of these metabolites were protective (*r*_*g*_ ≤ –0.13), except for the HDL-related traits → AN, which were risk-increasing (*r*_*g*_ ≥ 0.18; Table 1, Table S2). Sensitivity analyses supported four of these relationships despite the reduced statistical power: docosahexaenoic acid → ADHD ($\overline{{GCP}}$ = 0.61), cholesterol to total lipids ratio in large VLDL → ADHD ($\overline{{GCP}}$ = 0.72), total lipids in HDL → AN ($\overline{{GCP}}$ = 0.82) and free cholesterol to total lipids ratio in large VLDL → OCD ($\overline{{GCP}}$ = 0.53; Table S6).

### Putative causal relationships uncovered for HDL-related traits on AN

**Fig. 3:**
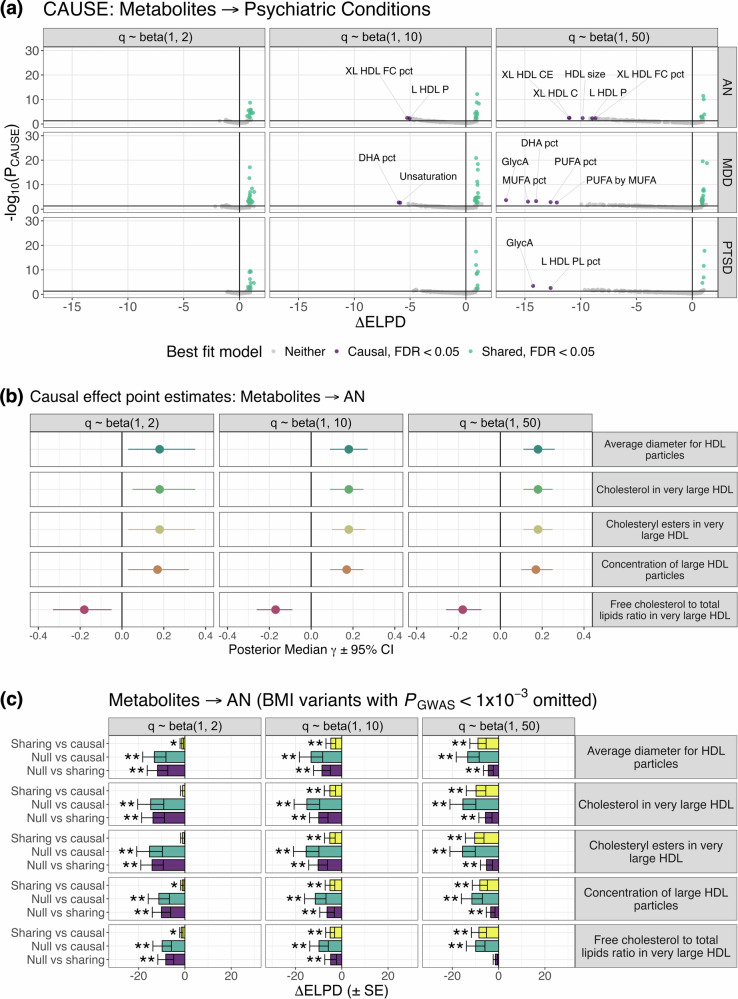
Causal modelling between metabolites and psychiatric illness using CAUSE. (**a**) Scatter plots summarising CAUSE models examining metabolites with evidence for a causal relationship with anorexia nervosa (AN), major depressive disorder (MDD) or post-traumatic stress disorder (PTSD). Each point represents a pairing of metabolite and psychiatric condition, noting these results are restricted to psychiatric conditions with evidence for at least one causal relationship. The x-axis corresponds to the change in expected log pointwise posterior density (*ΔELPD*) for CAUSE sharing and causal models (x-axis), with more negative values indicating causal models fit better than sharing models, and vice versa. The corresponding –log_10_(*P*) values are plotted on the left y-axis. Purple points = trait pairings with stronger evidence for causality at an *FDR*_*BH*_ < 0.05; green points = trait pairings with stronger evidence for horizontal pleiotropy at an *FDR*_*BH*_ < 0.05. Horizontal black line corresponds to a nominal *P* < 0.05. Facet headings indicate parameters used to define the prior beta distributions, with alpha = 1, beta = 2 representing the most conservative prior, and alpha =1, beta = 50 the most relaxed. Full results for all genetically correlated trait pairings identified via LDSR are available in Table S7 and S8. (**b**) Causal effect estimates (posterior median γ ± 95% credible interval [*CI*]) summarising direction of effect between all five HDL-related traits causally associated with AN. (**c**) *ΔELPD* (± standard error [*SE*]) estimates for the five HDL-related traits on AN after excluding variants associated with BMI [37] at a *P*_*GWAS*_ < 1 × 10^–3^. Yellow bars = the sharing versus causal model comparisons, similar to those depicted in panel (**a**); teal bars = comparison of causal and null models; purple bars = comparison of sharing and null models. Note that a negative *ΔELPD* estimate indicates that the second model (as indicated on the left y-axis) fits better than the first. * = *P* < 0.05, ** = *FDR*_*BH*_ < 0.05.

We further explored evidence for causality amongst genetically correlated traits using the CAUSE framework [34], which applies a Bayesian approach to Mendelian randomisation. Using prior distributions that assume moderate (q ~ beta(1,10)) or low (q ~ beta(1,50)) horizontal pleiotropy, five HDL-related traits exhibited stronger evidence for causality than horizontal pleiotropy with respect to AN (*FDR*_*BH*_ < 0.05, ΔELPD < 0; Fig. 3a, b, Table S7, S8). These include: average diameter for HDL particles, cholesterol/cholesteryl esters in very large HDL, concentration of large HDL particles, and free cholesterol to total lipids ratio in very large HDL. Although these trait pairings did not survive correction for multiple testing using the most conservative prior distribution (q ~ beta(1,2)), all ΔELPD estimates were directionally consistent (Fig. 3b, Table S7, S8). Reverse analyses utilising AN as the exposure trait revealed no evidence for reverse causality (Fig. S2, Table S9, S10). In contrast, MDD and PTSD exhibited evidence for bidirectional causal relationships with several metabolites, and thus were not analysed further (Fig. 3a, Fig. S2, Table S9, S10).

For most HDL-related traits → AN, the sign of the causal effect term (posterior median γ) was generally positive, indicating that elevation of each HDL trait is risk-increasing for AN (Fig. 3b, Table S8). The exception was free cholesterol to total lipids ratio in very large HDL, which was protective (Fig. 3b, Table S8). Given that DSM-IV (used for most cohorts in the AN GWAS) incorporates low BMI as a criterion for AN diagnosis [36], which may confound the HDL → AN causal estimates, we repeated these analyses after excluding all BMI-associated SNPs (*P*_*GWAS*_ < 1 × 10^–3^) per [37]. Strikingly, all causal relationships remained significant and directionally consistent (Fig. 3c, Fig. S3, Table S11, S12). However, sensitivity analyses using smaller metabolite GWAS [26] revealed no evidence for causality (Table S13, S14). This is likely since CAUSE is more sensitive to smaller sample sizes in these GWAS than LCV, as SNPs are preselected based on their association with the exposure trait (*P*_*GWAS*_ < 1 × 10^–3^).

Of the 12 significant trait pairings identified via LCV, 3 were nominally supported by CAUSE, specifically: total lipids in HDL → AN, free cholesterol in medium HDL → AN, and free cholesterol to total lipids ratio in very large HDL → PTSD (Table S15). In all cases, the causal effect term was directionally consistent with LCV and LDSC estimates (Table S15). For PTSD, we note there was weak evidence for reverse causality (*P* = 0.043; Table S15).

### Metabolites with psychiatric causality share genetic relationships with cortical structure

**Fig. 4:**
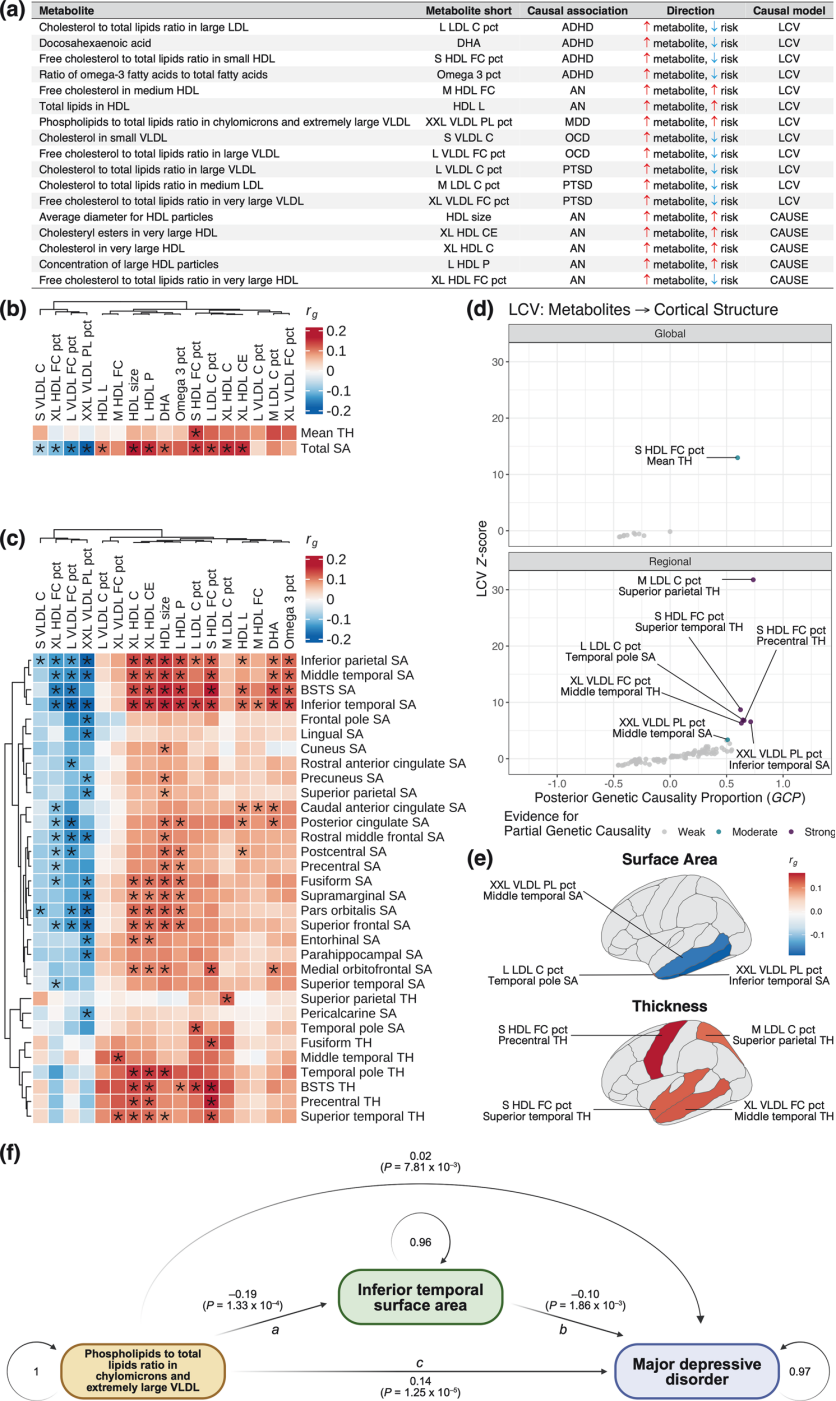
Genetic relationship between metabolites causally associated with psychiatric conditions and structure of the cerebral cortex. (**a**) Table summarising evidence for causal relationships between metabolites and psychiatric conditions. (**b**, **c**) Heatmaps presenting genetic correlation coefficients (*r*_*g*_) for the 17 metabolites reported in panel (**a**) with respect to mean cortical thickness (TH) and total surface area (SA) **(b)**, and region-specific measures of SA and TH (**c**). The regional results have been subset to only include regions with at least one significant genetic correlation. * = *FDR*_*BH*_ < 0.05. All columns and rows (regional measures only) have been subject to hierarchical clustering. (**d**) Scatter plots comparing LCV posterior genetic causality proportions ($\overline{{GCP}}$) to their corresponding *Z*-scores. Purple points = trait pairings with strong evidence for partial genetic causality ($\overline{{GCP}}$ ≥ 0.6); teal points = trait pairings with moderate evidence (0.5 ≤ $\overline{{GCP}}$ < 0.6). (**e**) Genetic correlation coefficients for trait pairings from panel (d) with strong or moderate evidence for causality, projected onto the left cerebral hemisphere (lateral view). (**f**) Genomic structural equation model testing whether inferior temporal surface area mediated the genetic association between phospholipids to total lipids ratio in chylomicrons and extremely large VLDL on major depressive disorder. Standardised pathway coefficients and their associated *P*-values are reported, as well as residual variances for each node. The indirect effect for this network (*a* x *b* = 0.019, *P* = 7.81 × 10^–3^) suggests partial mediation of the metabolite on major depressive disorder via cortical surface area, while the total effect (*c´* + *a* x *b* = 0.159, *P* = 4.07 × 10^–6^) indicates an overall positive association.

Alteration of cerebral anatomy is a common feature of many psychiatric conditions [38, 39]. We therefore examined genetic relationships between metabolites causally associated with psychiatric conditions and measures of the cerebral surface area and thickness using GWAS from the ENIGMA consortium [29]. We focussed on 12 metabolites with at least moderate evidence for partial genetic causality (LCV), and the five HDL-related traits associated with AN (CAUSE; Fig. 4a).

At an *FDR*_*BH*_ < 0.05, four metabolites displayed consistent negative genetic correlation with global and region-specific cortical surface area (*r*_*g*_ ≤ –0.08), with phospholipids to total lipids ratio in chylomicrons and extremely large VLDL most consistently represented (16 correlations; Fig. 4b, c, Table S16, S17). In contrast, 12 metabolites were positively correlated with surface area and thickness (*r*_*g*_ ≥ 0.07), with average diameter for HDL particles most frequently implicated (19 correlations; Fig. 4b, c, Table S16, S17).

LCV models revealed strong evidence for partial genetic causality between several metabolites and specific cortical regions, including: cholesterol to total lipids ratio in medium LDL → superior parietal thickness ($\overline{{GCP}}$ = 0.74), phospholipids to total lipids ratio in chylomicrons and extremely large VLDL → inferior parietal surface area ($\overline{{GCP}}$ = 0.71), and free cholesterol to total lipids ratio in small HDL → precentral thickness ($\overline{{GCP}}$ = 0.66), among others (Fig. 4d, Table S18, S19). Moderate evidence was also uncovered for free cholesterol to total lipids ratio in small HDL → mean cortical thickness ($\overline{{GCP}}$ = 0.597) and phospholipids to total lipids ratio in chylomicrons and extremely large VLDL → middle temporal surface area ($\overline{{GCP}}$ = 0.51; Fig. 4d, Table S18, S19). All relationships involving cortical thickness exhibited positive genetic correlation, suggesting elevation of these metabolites is associated with elevated thickness of these regions, whereas negative correlation was observed for surface area (Fig. 4c, e, Table S16, S17). One exception was cholesterol to total lipids ratio in large LDL → temporal pole surface area, which were positively correlated (Fig. 4c, e, Table S16, S17). Although CAUSE models did not identify significant evidence for causality, these findings nonetheless suggest some metabolites causally associated with psychiatric conditions may also influence cortical structure (Table S20–S23).

We additionally examined genetic relationships between these cortical measures and psychiatric traits to explore evidence for causal chains (i.e. metabolite → cortical measure → psychiatric trait). Several cortical regions were genetically correlated with MDD, ADHD and PTSD, but no evidence for causality was uncovered (Table S24–S27). We further employed genomic structural equation modelling to assess whether cortical structure mediates the relationship between these same metabolites and psychiatric traits [40]. Notably, the genetic relationship between phospholipids to total lipids ratio in chylomicrons and extremely large VLDL and MDD was partially mediated via inferior temporal surface area (*P*_*indirect*_ = 7.81 ×10^–3^*, P*_*total*_ = 4.07 × 10^–6^; Fig. 4f, Table S28). Interestingly, no evidence for mediation was uncovered for the remaining traits, suggesting any putative causal relationships between these metabolites and psychiatric conditions may not directly involve changes in cortical thickness or surface area (Table S28).

### Shared gene-level associations between metabolites and psychiatric conditions

**Fig. 5:**
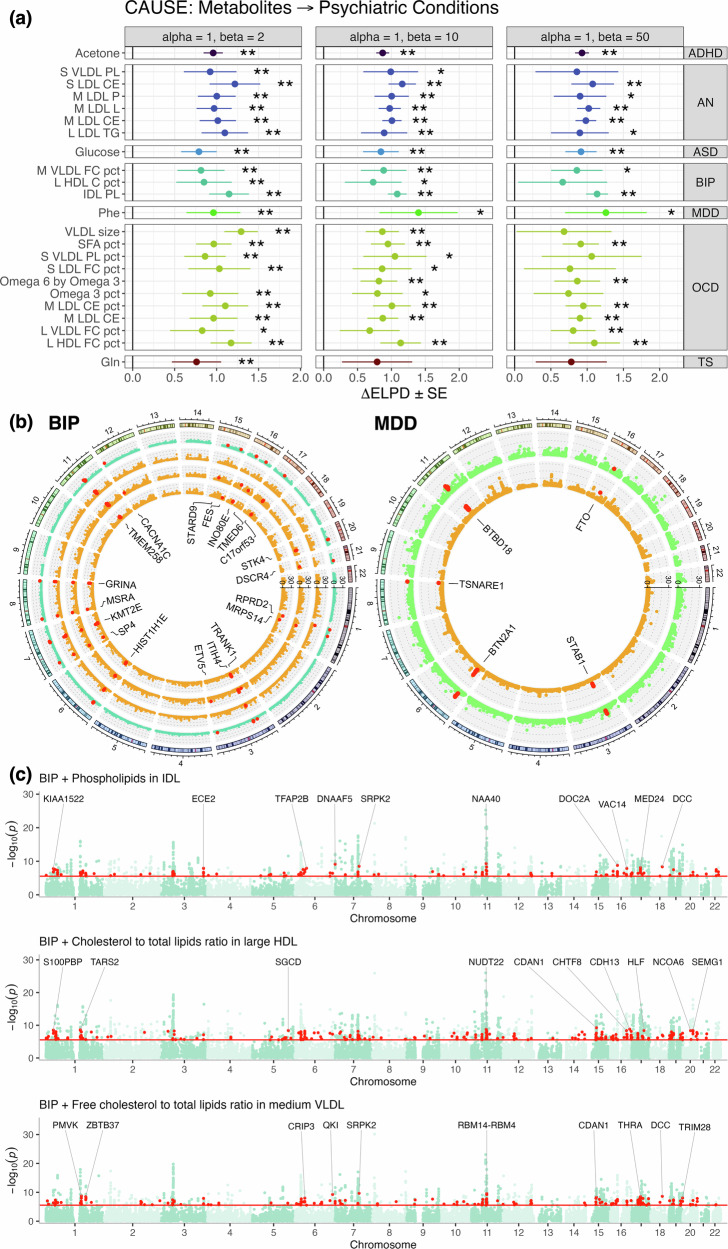
Shared gene-level common variant associations between metabolites and psychiatric illness. (**a**) Heatmap depicting *ΔELPD* estimates from the forwards CAUSE analysis (i.e. metabolite as exposure), subset to trait pairings that also exhibited nominal evidence for shared biology in the reverse analysis (i.e. psychiatric condition as exposure). * = *P* < 0.05, ** = *FDR*_*BH*_ < 0.05. (**b**) Circos plots presenting shared gene-level common variant associations for BIP, MDD and their respective associated metabolites presented in panel (**a**). Red points = genes with a Bonferroni-corrected *P*_*MAGMA*_ < 2.6 × 10^–6^ for the psychiatric trait and at least one metabolite. Each shell of each plot represents a different trait as follows, radiating inwards: (left) BIP, phospholipids in IDL, cholesterol to total lipids ratio in large HDL and free cholesterol to total lipids ratio in medium VLDL; (right) MDD and phenylalanine. (**c**) Manhattan plots depicting pairwise MAGMA gene-level meta-analyses for BIP and the same metabolites in panel (**b**). Each point represents a single gene, with –log_10_(*P*_*meta*_) from the meta-analyses plotted on the y-axis. Red points = genes that were nominally significant when each trait was analysed separately but surpassed a Bonferroni correction for multiple testing (*P* < 2.6 × 10^–6^, horizontal red line) in the meta-analysis.

To explore shared genetic architecture, we analysed metabolite-psychiatric trait pairings with stronger evidence for horizontal pleiotropy than causality, as identified by CAUSE (*FDR*_*BH*_ < 0.05, ΔELPD > 0; Fig. 3a, Table S7). Of these 137 pairings, 23 also showed nominal evidence for pleiotropy in reverse models and were thus prioritised for gene-level analysis (Fig. 5a, Table S29).

Using MAGMA, we identified genes significantly associated with both traits in each pairing (*P*_*Bonf*_ < 0.05). Notably, only BIP and MDD shared gene associations with their respective metabolites (Fig. 5b, Table S30). For BIP, 81 genes overlapped at least one of three metabolites, specifically: cholesterol to total lipids ratio in large HDL (65 genes), free cholesterol to total lipids ratio in medium VLDL (60) and Phospholipids in IDL (41; Fig. 5b, Table S30). These include genes involved in ion transport (e.g. *CACNA1C*, *KCNS1*, *SLC12A9*, *SLC4A1*), synaptic function (*ACHE*, *GLT8D1*, *GRINA*, *PACS1*, *TRANK1*, *TRIM38*), neuronal development and plasticity (*ETV5*, *MYRF*, *RCOR2*, *SP4*), metabolic function (*COX8A, FADS1*/*2*, *GAL3ST3*, *MSRA*), epigenetic regulation (including 10 histone proteins, *HDAC5*, *INO80E*, *KMT2E*, *PBRM1*) and immune function (*CTSF*, *FES*, *FEN1*, *ITIH1*, *ITIH3, ITIH4, STAB1*, TLR9, *TRIM38*, *WFDC5*; Fig. 5b, Table S30). We note these functional associations are not exhaustive or mutually exclusive.

MDD and phenylalanine shared 37 genes, including several that were identified in the analysis of BIP (e.g. *FADS1*/*2*, *GLT8D1*, *ITIH1*, *MYRF*, *PBRM1, TRIM38*). Among genes exclusively shared between MDD and phenylalanine, we identified regulators of neuronal development and function (e.g. *BTBD18*, *CLP1*, *CTNND1*, *TSNARE1*, *ZDHHC5*), and immune function (*BTN2A1*, *BTN3A2*, *SERPING1*). Another notable shared association was the fat mass and obesity-associated gene *FTO*, a brain-enriched RNA N6-methyladenosine demethylase associated with energy homeostasis, satiety and obesity [41]. Despite these overlapping associations, no shared canonical pathways (Molecular Signatures Database, 3,917 pathways) were identified via a gene-set analysis after correction for multiple testing (*FDR*_*BH*_ < 0.05), suggesting that shared gene-level associations may reflect diverse biological mechanisms.

### Pairwise meta-analyses reveal novel gene and pathway associations between metabolites and psychiatric traits

To identify novel shared biology, we conducted pairwise meta-analyses of gene-level associations for the 23 metabolite-psychiatric trait pairings with evidence of shared genetic architecture. Genes nominally significant in the MAGMA analyses (*P*_*Bonf*_ < *P* < 0.05) were meta-analysed using Stouffer’s method.

Novel gene associations were identified for all pairings (Fig. 5c, Fig. S4, Table 2, Table S31). BIP and cholesterol to total lipids ratio in large HDL yielded the most (*N*_*novel*_ = 263, top hit = *CDAN1*, *P*_*meta*_ = 5.74 × 10^–10^), with BIP also showing the highest number of novel genes per metabolite (203 genes per metabolite). Strikingly, genes related to neuronal development and function were consistently identified in association with BIP and its corresponding metabolites, including *CACNB2*, *CDH13*, *DOC2A*, *HOMER2*, *NRCAM*, *S100PBP*, *SLC29A2*, *SNAP91* (Fig. 5d, Table S31). Notably, the dopamine receptor *DRD2*, a key psychotropic drug target, was also represented in the meta-analysis of BIP/free cholesterol to total lipids ratio in medium VLDL (Table S31).

**Table 2:** Number of novel genic associations between metabolites and psychiatric conditions after MAGMA gene-level meta-analysis.

Psychiatric condition	Metabolite	*N*_*novel associations*_ *(P*_*Bonf*_ < 0.05)	Top gene	*P*_*meta*_
**ADHD**	Acetone	23	*PITPNM2*	9.93 × 10^–9^
**AN**	Cholesteryl esters in medium LDL	35	*ODF4*	6.92 × 10^–8^
	Cholesteryl esters in small LDL	47	*ARHGEF15*	1.64 × 10^–8^
	Concentration of medium LDL particles	38	*ARHGEF15*	9.1 × 10^–8^
	Phospholipids in small VLDL	50	*LGR4*	4.64 × 10^–8^
	Total lipids in medium LDL	45	*ALDH18A1*	1.25 × 10^–7^
	Triglycerides in large LDL	42	*SLC2A4*	5.67 × 10^–8^
**ASD**	Glucose	17	*SV2A*	1.66 × 10^–7^
**BIP**	Cholesterol to total lipids ratio in large HDL	263	*CDAN1*	5.74 × 10^–10^
	Free cholesterol to total lipids ratio in medium VLDL	204	*SRPK2*	2.13 × 10^–10^
	Phospholipids in IDL	143	*NAA40*	4.88 × 10^–10^
**MDD**	Phenylalanine	70	*ACO1*	1.42 × 10^–9^
**OCD**	Average diameter for VLDL particles	17	*MTCH2*	7.42 × 10^–7^
	Cholesteryl esters in medium LDL	5	*CORO1B*	2 × 10^–6^
	Cholesteryl esters to total lipids ratio in medium LDL	13	*NDUFS3*	6.23 × 10^–7^
	Free cholesterol to total lipids ratio in large HDL	12	*GAK*	1.04 × 10^–6^
	Free cholesterol to total lipids ratio in large VLDL	4	*TRIT1*	5.3 × 10^–7^
	Free cholesterol to total lipids ratio in small LDL	16	*KIT*	2.11 × 10^–7^
	Phospholipids to total lipids ratio in small VLDL	6	*MCPH1*	6.6 × 10^–7^
	Ratio of omega-3 fatty acids to total fatty acids	8	*KCND2*	6.44 × 10^–7^
	Ratio of omega-6 fatty acids to omega-3 fatty acids	10	*RAB40C*	8.08 × 10^–7^
	Ratio of saturated fatty acids to total fatty acids	1	*DRG2*	5.84 × 10^–7^
**TS**	Glutamine	24	*RANBP10*	5.44 × 10^–7^

Other trait pairings also revealed functionally relevant genes, including *SV2A* (ASD/glucose), *MCPH1* (OCD/Phospholipids to total lipids ratio in small VLDL), *KCND2* (OCD/Ratio of omega-3 fatty acids to total fatty acids) and *RAB40C* (OCD/Ratio of omega-6 fatty acids to omega-3 fatty acids; Table 2). Many of the top associations were also related to metabolic function, including: *PITPNM2* (ADHD/acetone), *LGR4* (AN/phospholipids in small VLDL), *ALDH18A1* (AN/total lipids in medium LDL), *SLC2A4* (AN/triglycerides in large LDL), *NAA40* (BIP/phospholipids in IDL), *ACO1* (MDD/phenylalanine), *NDUFS3* (OCD/Cholesteryl esters to total lipids ratio in medium LDL) and *TRIT1* (OCD/Free cholesterol to total lipids ratio in large VLDL).

A similar gene-set meta-analysis uncovered 28 significant pathways (*FDR*_*BH*_ < 0.05) across 10 trait pairings. BIP and cholesterol to total lipids ratio in large HDL returned the largest number of gene-set associations (11 novel pathways), including the *MAPK pathway*, *Brain-derived neurotrophic factor (BDNF) signalling*, *Insulin signalling* and *Serotonin receptor 4/6/7 and NR3C signalling*, among others (Table 3). This was followed by AN/triglycerides in large LDL, which shared: *Leptin pathway, Obesity pathway*, *Abacavir transmembrane transport* and *MET receptor activation* gene-sets, and ASD/glucose, which shared the *SMAD2/3 pathway*, *Hemostasis* and *Opioid receptor pathways* (Table 3). Overall, these results reveal novel gene-level common variant associations shared between psychiatric conditions and metabolites, that in some cases converge on significant pathways.

**Table 3:** MAGMA pathway-level meta-analysis.

Psychiatric condition	Metabolite	Pathway	Source	*N*_*genes*_^a^	*β*^b^	*SE*^c^	*P*^d^	*FDR*_*BH*_^e^
**ADHD**	Acetone	MECP2 regulates transcription factors	Reactome	4	1.881	0.511	1.17 × 10^–4^	0.049
		Disorders in ketone body synthesis	WikiPathways	5	1.387	0.379	1.26 × 10^–4^	0.049
**AN**	Cholesteryl esters in medium LDL	Macrophage-stimulating protein MSP signalling	WikiPathways	104	0.410	0.120	3.18 × 10^–4^	0.031
		TROP2 regulatory signalling	WikiPathways	48	0.602	0.182	4.60 × 10^–4^	0.042
	Total lipids in medium LDL	LDL pathway	BioCarta	6	1.906	0.553	2.83 × 10^–4^	0.034
		Macrophage-stimulating protein MSP signalling	WikiPathways	104	0.404	0.120	3.92 × 10^–4^	0.043
	Triglycerides in large LDL	Leptin pathway	BioCarta	11	1.229	0.383	6.69 × 10^–4^	0.045
		Obesity pathway	BioCarta	7	1.721	0.509	3.60 × 10^–4^	0.029
		Abacavir transmembrane transport	Reactome	5	1.999	0.632	7.77 × 10^–4^	0.050
		MET receptor activation	Reactome	6	1.798	0.548	5.18 × 10^–4^	0.037
**ASD**	Glucose	SMAD2/3 pathway	PID	16	0.905	0.247	1.24 × 10^–4^	0.023
		Hemostasis	Reactome	556	0.167	0.045	1.08 × 10^–4^	0.022
		Opioid receptor pathways	WikiPathways	39	0.603	0.179	3.79 × 10^–4^	0.049
**BIP**	Cholesterol to total lipids ratio in large HDL	ARENRF2 pathway	BioCarta	19	1.031	0.270	6.88 × 10^–5^	0.014
		MAPK pathway	BioCarta	76	0.563	0.128	5.79 × 10^–6^	0.003
		MAPK signalling pathway	KEGG	246	0.279	0.075	9.27 × 10^–5^	0.017
		CREB phosphorylation	Reactome	6	1.762	0.474	1.02 × 10^–4^	0.018
		Signalling by NTRKs	Reactome	130	0.415	0.101	2.21 × 10^–5^	0.007
		7q11.23 copy number variation syndrome	WikiPathways	86	0.453	0.124	1.38 × 10^–4^	0.020
		Brain-derived neurotrophic factor BDNF signalling	WikiPathways	132	0.381	0.104	1.24 × 10^–4^	0.019
		Host-pathogen interaction of human coronaviruses MAPK signalling	WikiPathways	34	0.767	0.203	7.89 × 10^–5^	0.015
		Insulin signalling	WikiPathways	153	0.322	0.092	2.41 × 10^–4^	0.033
		Serotonin receptor 4/6/7 and NR3C signalling	WikiPathways	17	1.112	0.290	6.25 × 10^–5^	0.014
		VEGFA/VEGFR2 signalling	WikiPathways	395	0.203	0.060	3.26 × 10^–4^	0.042
	Free cholesterol to total lipids ratio in medium VLDL	7q11.23 copy number variation syndrome	WikiPathways	86	0.451	0.119	7.94 × 10^–5^	0.022
	Phospholipids in IDL	CDMAC pathway	BioCarta	15	1.107	0.307	1.52 × 10^–4^	0.035
**OCD**	Cholesteryl esters to total lipids ratio in medium LDL	Clock-controlled autophagy in bone metabolism	WikiPathways	77	0.423	0.129	5.16 × 10^–4^	0.047
**TS**	Glutamine	Medicus pathogen EBV EBNA1 to p53 mediated transcription	KEGG	8	1.677	0.462	1.44 × 10^–4^	0.025

^a^The number of genes within each listed pathway.

^b^The effect size estimate summarising the association between the pathway and trait pairing of interest.

^c^Standard error of the effect size estimate.

^d^P-value testing whether the effect size estimate is significantly different from zero.

^e^The Benjamini-Hochberg false discovery rate.

## Discussion

Metabolites related to cardiometabolic health are frequently dysregulated in psychiatric conditions, yet their causal relevance to psychiatric health remains unclear. In this study, we systematically examined genetic correlation and causality between 249 circulating metabolites and 10 psychiatric conditions, identifying trait-specific biological relationships. We observed widespread genetic correlation between psychiatric illness and lipid traits, particularly fatty acids, cholesterol and lipoprotein traits. This is consistent with observational and genetic evidence indicating that dyslipidaemia is a common feature of many psychiatric conditions, wherein alteration of lipid profiles is thought to impact variables such as inflammation, neuronal structure and neurotransmission [27, 28, 42–45]. Amino acids (e.g. glutamine, tyrosine) and glycolysis-related traits (e.g. glucose, citrate) also showed associations, aligning with studies linking these metabolites with psychiatric health [10, 46–50]. Glycaemic traits, notably, are strongly comorbid with SZ independent of medication effects [51, 52], and dysregulation of amino acids related to neurotransmission have been implicated in both MDD and SZ [53–55]. Despite extensive correlation, many metabolites did not show evidence for causality. This conflicts with recent Mendelian randomisation studies that have reported evidence for causality for some of these metabolites, including polyunsaturated fatty acids, triglycerides and glucose [43, 46, 56]. This discrepancy may reflect methodological differences. For instance, CAUSE accounts for correlated pleiotropy and confounding, potentially reducing false positives [34]. Furthermore, our use of the largest uniformly processed metabolite GWAS to date [25] likely improved power to clarify these relationships. Nonetheless, pleiotropic effects may still mediate associations, suggesting these metabolites remain promising biomarkers or therapeutic targets.

We found robust evidence that elevation of specific HDL traits, particularly particle diameter, concentration and cholesterol content, is causally associated with increased odds of AN. These findings build upon previous observational studies reporting elevated blood HDL concentrations amongst individuals with AN [57, 58], as well as genetic studies reporting positive genetic correlation between these variables [59]. We consider our findings to be particularly robust since evidence for causality was retained after removing variants associated with BMI, addressing a key diagnostic confounder [36]. While the biological mechanism linking these HDL-related traits to AN risk remains unclear, genetic correlation between HDL traits and structural properties of cortical regions previously implicated in AN (e.g. superior frontal, insula, precuneus) suggests potential neuroanatomical mediation [60–62], necessitating deeper analysis to deconvolute association among these traits. Notably, one HDL trait (free cholesterol in very large HDL) was associated with decreased odds of AN, warranting further investigation to ascertain the biological significance of this relationship. In contrast to these causal relationships, we also highlight that shared gene-level associations were also identified between AN and LDL-/VLDL-related traits, suggesting a particularly complex relationship between circulating lipoproteins and AN risk.

We also identified a causal, risk-increasing relationship for phospholipid ratios in chylomicrons and extremely large VLDL on MDD. Although prior studies have reported dyslipidaemia as a feature of MDD [42, 43, 63], evidence specifically implicating phospholipids, chylomicrons and/or VLDL is limited and conflicting [64–66], highlighting the need for further validation. Additionally, elevated LDL and/or VLDL cholesterol content exhibited evidence for a protective effect in OCD and PTSD, however few studies have examined these specific traits in either condition. For PTSD, observational studies have consistently reported elevation of total cholesterol and LDL among individuals with this condition [67, 68], but it is unclear whether the cholesterol content of LDL is specifically altered. There is also limited evidence pertaining to dysregulation of VLDL in OCD, with some observational evidence suggesting serum VLDL is elevated [69], while other studies have reported little-to-no association [70]. We therefore emphasise the need for further investigation of these traits to clarify association, dissect correlation from causation, and identify specific features of LDL/VLDL with potential clinical utility in OCD and PTSD.

In ADHD, we uncovered moderate evidence for protective effects of docosahexaenoic acid (DHA), omega-3 fatty acid ratios, and cholesterol content of small HDL and large LDL. For DHA and omega-3 fats, these findings align with randomised controlled trials [71–74] and observational evidence [75, 76] supporting beneficial effects in ADHD. Evidence for LDL and HDL traits is mixed, with some studies suggesting these traits are altered in the serum of affected individuals [77, 78], while others report limited association [79, 80]. However, our causal modelling suggests these lipoprotein traits enhance structural properties of cortical brain regions previously associated with ADHD [81–84], such as precentral and superior temporal thickness and temporal pole surface area. Although genomic structural equation modelling did not support mediation via neuroanatomical changes, future multivariable causal modelling incorporating larger and longitudinal neuroimaging GWAS may help clarify whether these putative protective effects are mediated by brain morphology [85].

Beyond causal relationships, we identified extensive gene-level overlap between metabolites and psychiatric conditions, particularly in trait pairings with strong evidence for horizontal pleiotropy. BIP and lipoprotein traits shared numerous genes involved in neuronal development, synaptic function, metabolism, epigenetic regulation and immune responses. For instance, *FADS1/2* have been recently implicated in a putative causal relationship between BIP and arachidonic acid [56] and prioritised for drug repurposing [9], with our results further suggesting these genes may also link BIP with lipoprotein-related traits. Genes associated with synaptic function (e.g. *CACNB2*, *HOMER2*, *NRCAM*, and *SNAP91*) that may contribute to neural circuit dysregulation in BIP [86] were also related to lipoprotein traits, suggesting functional convergence. In this case, it is possible that lipoprotein traits are interrelated with biological processes such as synaptic vesicle release that are also dysregulated in BIP [86]. We further identified shared variant signatures at *DRD2* between BIP and free cholesterol ratios in medium VLDL. Given *DRD2* is a key target of psychotropic medications, this overlap may reflect known metabolic side effects or shared biological pathways [87, 88]. Supporting this, recent work shows DRD2-expressing neurons can be modulated by lipoprotein lipase LPL, which itself responds to dietary triglycerides [89]. These findings highlight the potential for metabolite-gene interactions to inform pharmacogenomics and treatment stratification.

In summary, our results present evidence for causal relationships and shared genetic architecture between circulating metabolites and psychiatric conditions with potential utility for clinical management. There are some limitations that need to be considered when interpreting these results. Firstly, the analyses conducted in this study are subject to inherent limitations of the GWAS data, such as population stratification [90] and other biases. For example, the UK Biobank is composed of individuals over the age of 40, thus this age bias may have affected GWAS for the metabolic traits [91]. Similarly, our analyses were restricted to individuals of European ancestry, limiting the applicability of our findings to other populations. While we prioritised metabolite GWAS with the greatest available sample size to maximise statistical power, we also note that further investigation with more-dense metabolite panels (e.g. [92]) could provide greater biological resolution at the expense of power. All trait pairings with evidence for a causal relationship also require validation via randomised controlled trials. While we attempted replication using independent metabolite GWAS [26], CAUSE results were inconsistent, likely due to reduced sample size. Nonetheless, CAUSE methodology offers a strong framework for causal inference, mitigating confounding from horizontal pleiotropy and sample overlap [34]. Finally, our exploration of brain regions causally associated with metabolites was not exhaustive, and future work should include additional brain structures (e.g. subcortical structures), and measures (e.g. connectivity) relevant to psychiatric illness [93]. Overall, this study provides a comprehensive genetic atlas of metabolite-psychiatric trait relationships, highlighting specific metabolites with potential causal roles and shared biology. These findings offer a foundation for future research into metabolic contributions to psychiatric risk and treatment response.

## Supplementary information

Supplementary methods and figures

Supplementary Tables

## Data Availability

All GWAS summary statistics utilised in this study are publicly available and can be accessed via the Psychiatric Genomics Consortium website (https://pgc.unc.edu/for-researchers/download-results/) or the GWAS Catalog with accession numbers GCST90451106–GCST90451354 [25], and GCST90301941–GCST90302173 [26]. Sample scripts used in the present study can be accessed at: https://github.com/D-Kiltschewskij/Genetic_Correlation_Causation_Metabolic_Psychiatric_Traits.
