# Supplementary material for: A genetic atlas of relationships between circulating metabolites and liability to psychiatric conditions
Source: Mol Psychiatry. 2026 Feb 6;31(6):3345–59. doi: 10.1038/s41380-026-03464-z (PMC13190307; doi:10.1038/s41380-026-03464-z)
Supplement: Supplementary file 1 — Supplementary methods and figures [file 41380_2026_3464_MOESM1_ESM.docx]

**A Genetic Atlas of Relationships Between Circulating Metabolites and Liability to Psychiatric Conditions**

**Supplementary Methods and Figures**

**Contents**

**Page 2–7 SUPPLEMENTARY METHODS.**

**Page 8–10. Figure S1.** Genetic correlation among psychiatric conditions and traits related to lipoprotein subclasses.

**Page 11. Figure S2.** Reverse CAUSE models examining the effect of psychiatric traits on metabolites.

**Page 12. Figure S3.** CAUSE posterior gamma estimates for HDL-related traits on AN, with BMI-associated SNPs excluded.

**Page 13–18. Figure S4.** Manhattan plots for all MAGMA gene-level meta-analyses.

**Page 19–20. REFERENCES**.

**SUPPLEMENTARY METHODS**

**Study overview**

The primary aim of this work was to better understand how clinically actionable metabolites impact psychiatric health by systematically exploring the genetic relationship between these traits using SNPs (Fig. 1). We specifically leveraged the largest, uniformly processed metabolite GWAS available to explore genetic correlation, followed by genetic causal inference using Latent Causal Variable (LCV) models and the Causal Analysis Using Summary Effect estimates (CAUSE) methodology. The goal of this analysis was to uncover metabolites with evidence for a causal effect on psychiatric conditions and therefore generate a resource that prioritises these metabolites for further investigation via clinical trials. In addition, the CAUSE methodology was also used to identify trait pairings with stronger evidence for shared genetic architecture, rather than a causal relationship. These traits were specifically analysed for shared, gene level common variant signatures using the Multimarker Analysis of GenoMic Annotation (MAGMA), with a view of both identifying common genes and mechanisms that may mediate the interplay between metabolic and psychiatric health.

**GWAS summary statistics**

GWAS summary statistics for 10 psychiatric conditions were obtained from the Psychiatric Genomics Consortium (PGC), prioritising the largest available GWAS of predominantly European ancestry. We specifically examined attention deficit hyperactivity disorder (ADHD, *N* = 225,534, [1]), anorexia nervosa (AN, *N* = 72,517, [2]), autism spectrum disorders (ASD, *N* = 46,351, [3]), bipolar disorder (BIP, *N* = 840,309, [4]), major depressive disorder (MDD, *N* = 2,000,702, [5]), obsessive compulsive disorder (OCD, *N* = 9,725, [6]), panic disorder (PD, *N* = 9,907, [7]), post-traumatic stress disorder (PTSD, *N* = 1,249,840, [8]), schizophrenia (SZ, *N* = 130,644, [9]) and Tourette syndrome (TS, *N* = 14,307, [10]).

GWAS summary statistics for 249 metabolites were obtained from the large, uniformly processed meta-analysis (*N* = 599,529) of 185,352 individuals from the Estonian Biobank and 413,897 individuals of European ancestry from the UK Biobank, conducted by [11]. The metabolites include triglycerides, fatty acids, phospholipids, cholesterol, ketone bodies, glycolysis related metabolites and amino acids, amongst others. All metabolites were measured in EDTA plasma samples via nuclear magnetic resonance (NMR) and inverse normal transformed prior to within-cohort association testing, using the sex, age, age^2^ and genetic principal components (top 10 for EstBB, top 20 for UKB) as covariates. For all metabolite-psychiatric trait pairings with significant evidence for causality, sensitivity analyses were conducted using an independent metabolite GWAS from [12], a meta-analysis of 136,016 individuals across 33 independent (non-Estonian or UK Biobank) cohorts. GWAS of structural measures of the cerebral cortex were obtained from the ENIGMA consortium [13]. We specifically used GWAS of global and regional surface area and thickness, generated from a meta-analysis of 33,992 individuals of predominantly European ancestry across 49 cohorts. All data were covaried for age, age^2^, sex, sex-by-age and age^2^ interactions, the first four genetic multidimensional scaling components, diagnostic status and scanner. For all summary statistics, we excluded 1) palindromic variants, 2) non-SNP variants (e.g. insertions, deletions), 3) variants with low imputation accuracy, where available (INFO < 0.9).

**Genetic correlation**

Genetic correlation was estimated between all metabolites and psychiatric conditions via linkage disequilibrium score regression (LDSR), as implemented in the *ldsc* package (v1.0.1) [14, 15]. Briefly, LDSR estimates genetic covariance between traits by regressing SNP level *χ*^2^ values – the product of SNP *Z*-scores from each trait – against LD scores that estimate total LD for a given SNP. Genetic covariance is then normalised to trait heritabilities to obtain genetic correlation (*r_g_*), noting that an intercept term is included that mitigates confounding due to factors such as sample overlap between GWAS.

All summary statistics were firstly harmonised into a “munged” format, wherein SNP effect sizes and standard errors were transformed into *Z*-scores, ensuring the sign of each *Z*-score was relative to the statistical effect allele. We also retained approximately 1 million high-confidence HapMap3 SNPs outside the major histocompatibility complex region (MHC, chr6:28000000–34000000) with minor allele frequency (MAF) > 0.05. Genetic correlation was then estimated using the *ldsc.py* script, with LD scores sourced from the 1000 Genomes Project European reference panel (available at: https://alkesgroup.broadinstitute.org/LDSCORE/). Trait pairings surpassing a Benjamini-Hochberg false discovery rate (*FDR_BH_*) < 0.05 were considered significant and retained for causal inference.

**Latent causal variable modelling**

Evidence for causality was assessed amongst genetically correlated trait pairings using a Latent Causal Variable model (LCV, [16]). LCV assumes a latent variable, *L*, mediates the genetic correlation between two traits. As such, if trait 1 has stronger genetic correlation with *L* than trait 2, trait 1 is deemed to be partially genetically causal for trait 2. Partial genetic causality is quantified as a posterior genetic causality proportion ($\hat{GCP}$), derived by comparing the mixed fourth moments (co-kurtosis) of SNP marginal effect size distributions between two traits. This leverages the fact that if trait 1 is partially genetically causal for trait 2, most SNPs affecting trait 1 will have proportional effects on trait 2 but not vice versa. $\hat{GCP}$ estimates between 0 (no genetic causality) and ±1 (full genetic causality) summarise the strength of evidence for a causal relationship for trait 1 on trait 2 and strictly do not serve as a magnitude of causal effect. Positive $\hat{GCP}$ values indicate evidence that trait 1 is partially genetically causal for trait 2, whereas negative values suggest trait 2 exhibits evidence for causality on trait 1. As shown previously, trait pairings with |$\hat{GCP}$| estimates ≥ 0.6, directionally consistent *Z*-scores and *FDR_BH_* < 0.05 were considered to exhibit strong evidence for partial genetic causality [16]. Trait pairings fulfilling these criteria with 0.5 ≤ |$\hat{GCP}$| < 0.6 were deemed to have moderate evidence for partial genetic causality. All summary statistics were munged prior to analysis as recommended [17].

**CAUSE**

In addition to LCV, the Causal Analysis Using Summary Effect estimates (CAUSE) method (v1.2.0.0335, [18]) was also used to estimate causal relationships amongst genetically correlated traits. Briefly, CAUSE is a method of Mendelian randomisation (MR) that uses SNPs associated with an exposure (i.e. metabolites) as instrumental variables (IVs) to explore evidence for causality with respect to an outcome trait (i.e. psychiatric conditions). CAUSE specifically uses a Bayesian framework that estimates causal effects conditioned on confounding pleiotropy (i.e. IVs acting directly on the outcome or via a confounding variable) by employing a beta prior distribution. This approach is applied to independent SNPs that are broadly associated with the exposure (*P*_GWAS_ < 1 x 10^–3^) to simultaneously capture causal relationships, correlated pleiotropy and uncorrelated pleiotropy. The ability to condition on correlated pleiotropy is particularly advantageous, as this cannot be readily assessed using conventional tests for pleiotropy (e.g. Cochran’s *Q* test, Egger intercept). Unlike many other summary-based MR models, CAUSE also includes a correlation term that empirically estimates and adjusts for sample overlap between the exposure and outcome and other confounders.

For all genetically correlated trait pairings, we used CAUSE to specifically compare models of genetic causality with horizontal pleiotropy against sharing models that only examine horizontal pleiotropy by fixing the causal effect (γ) at zero. These models were assessed using three distinct beta prior distributions, which assume high (⍺ = 1, β = 2), moderate (⍺ = 1, β = 10, default prior) or low correlated pleiotropy (⍺ = 1, β = 50), the former representing the most conservative but least powered model, while the latter is the most well-powered (and similar to traditional summary-based MR), but can be susceptible to false positives. Model comparisons were expressed as a change in expected log pointwise posterior density (*ΔELPD*), wherein negative values suggest the causal model fits better than sharing the sharing model, while positive values suggest the sharing model provides the best fit. This was examined for trait pairings with Pareto *k* estimates < 0.67, whereas those with Pareto *k* ≥ 0.67 were excluded from further analyses. Metabolite-psychiatric trait pairings with significant evidence for causality were defined as those with a negative *ΔELPD* that surpassed an *FDR_BH_* < 0.05, and also exhibited no evidence for causality in reverse models that used the psychiatric trait as the exposure and metabolite as outcome. We also define trait pairings with significant evidence for shared genetic architecture as those with a positive *ΔELPD* that surpassed an *FDR_BH_* < 0.05 in the forwards analysis, and *P* < 0.05 in the reverse analyses.

**Genomic structural equation modelling (SEM)**

Genomic SEM [19] was employed to investigate whether genetic effects on cortical structure mediate the relationship between circulating metabolites and psychiatric conditions. This analysis was restricted to metabolites with significant evidence for a causal relationship with both a cortical measure and psychiatric condition. GWAS summary statistics for each trait were munged and restricted to HapMap3 high confidence SNPs per the LDSC and LCV analyses. For each combination of metabolite, cortical measure and psychiatric trait, LDSC was used to estimate the genetic covariance matrix (S) and sampling covariance matrix (V) among the three traits, which served as input to the SEM.

Population and sample prevalence estimates for psychiatric traits were incorporated during LDSC to convert observed-scale SNP-heritability estimates to the liability scale. We specifically assumed population prevalences as follows: ADHD: 5% [1], MDD: 15% [20], PTSD: 10% [21].

A mediation model was specified such that the genetic component of a metabolite (M) influenced a cortical measure (C), which in turn influenced a psychiatric condition (P), as follows:

1. $C=aM+e_{c}$

2. $P=bC+c^{'}M+e_{p}$

Where;

*a* = the genetic path from the metabolite to cortical structure.

*b* = the genetic path from cortical structure to psychiatric condition.

*c´* = the direct path from the metabolite to the psychiatric condition, controlling for cortical structure.

The indirect effect of the metabolite on the psychiatric trait through cortical structure was calculated as *a* x *b*, and the total effect as *c´* + (*a* x *b*). All paths were estimated using the *genomicSEM R* package (v0.0.5) [19], with standard errors derived from the delta method.

**Gene and gene-set association analysis**

Common variant signatures for metabolites or psychiatric conditions were aggregated within protein coding genes using the Multimarker Analysis of GenoMic Annotation (MAGMA, v1.10, Linux, [22]). This analysis was restricted to all trait pairings with evidence for shared biology as nominated by CAUSE, with the aim of comparing shared gene and gene-set associations between each nominated trait pairing. GWAS SNPs were firstly mapped to 19,240 autosomal protein-coding genes in the GRCh37 genome assembly (NCBI), available at: <https://vu.data.surfsara.nl/index.php/s/Pj2orwuF2JYyKxq>. Genic boundaries were extended to 5kb upstream and 1.5kb downstream to include potential regulatory variants, and furthermore, we excluded genes within the MHC region due to the complexity of LD in this region. Gene-level association was then assessed via the default gene-based test in MAGMA, which uses a linear combination of SNP-level GWAS *P-*values to produce mean *χ^2^* test-statistics for each gene. To account for dependent *P*-values due to LD between variants, the 1000 genomes phase 3 European reference panel was used to derive variant-level LD, which weights the contribution of each SNP to the final association test-statistic. Gene associations with a Bonferroni-corrected *P* < 2.6 x 10^–6^ were deemed statistically significant, adjusting for 19,240 independent tests for each phenotype.

The MAGMA competitive gene-set association test was then employed to identify canonical pathways from the Molecular Signatures Database (MSigDB, 3917 pathways) associated with each phenotype of interest. MAGMA specifically constructs linear regression models that test whether genic association (transformed to *Z*-scores via the probit function) is stronger within the gene set of interest, compared to all other genes, with covariates for gene size and gene minor allele count. Gene-set associations with *FDR_BH_* < 0.05 were deemed significant.

**Pairwise gene and gene-set meta-analysis**

Genic *Z*-scores for metabolites and psychiatric traits with evidence for shared biology were subjected to pairwise meta-analyses using the --meta flag in MAGMA. This utilises Stouffer’s weighted *Z* method, which combines *Z*-scores for each gene (*i*) weighted by the GWAS sample size (*ω_i_*) as follows:

$$Z_{meta} \sim\frac{\sum_{i=1}^{k} \omega_{i}Z_{i}}{\sqrt{\sum_{i=1}^{k} \omega_{i}}}$$

To mitigate bias due to sample overlap and other confounders, the LDSR intercept for the metabolite and psychiatric condition of interest was included as a covariate as recommended by the MAGMA authors. Gene-set meta-analyses were additionally undertaken using the model described above, with *Z_meta_* as the outcome variable in each instance.


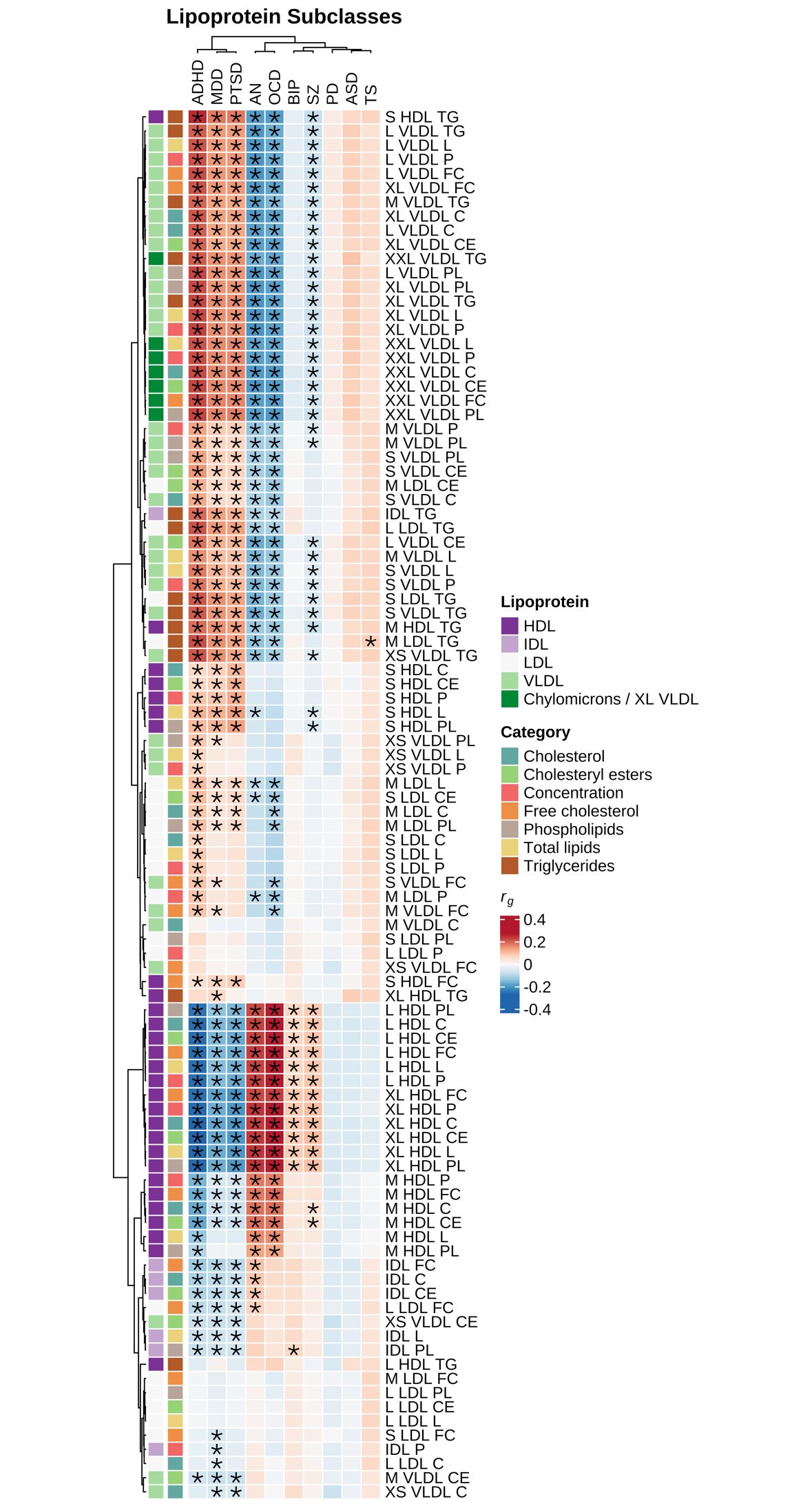


**(a)**

**Lipoprotein absolute lipid content**


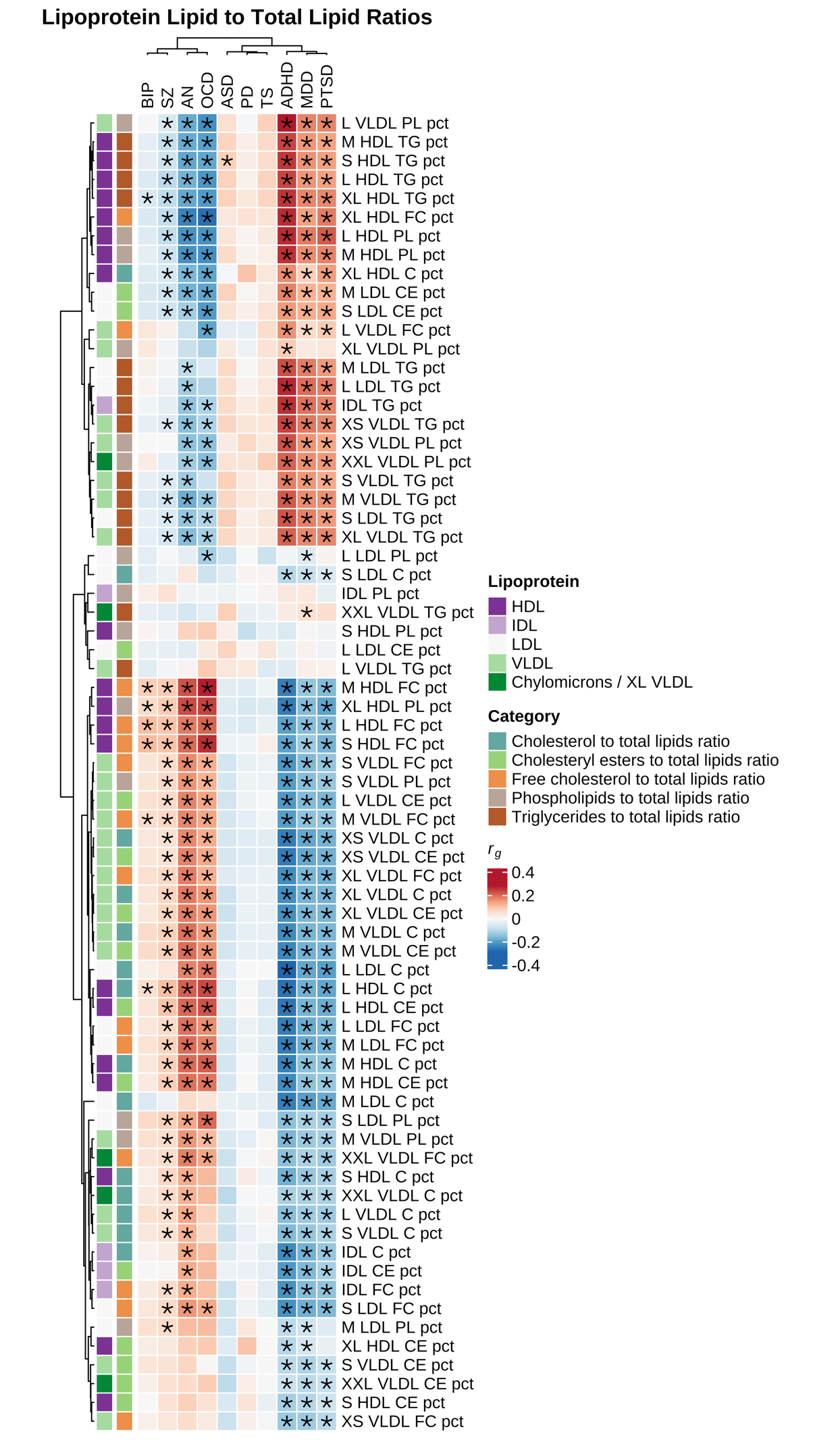


**(b)**

**Lipoprotein lipid ratios**

**Figure S1. Genetic correlation among psychiatric conditions and traits related to lipoprotein subclasses.** Heatmaps depicting LDSR genetic correlation coefficients (*r_g_*) between the 10 psychiatric conditions, **(a)** lipoprotein absolute lipid content and **(b)** lipoprotein lipid ratios, subset by lipoprotein particle diameter. Rows and columns were subject to hierarchical clustering to identify similar groups of traits. **FDR_BH_* < 0.05. Full metabolite names can be accessed in Table S1.

**
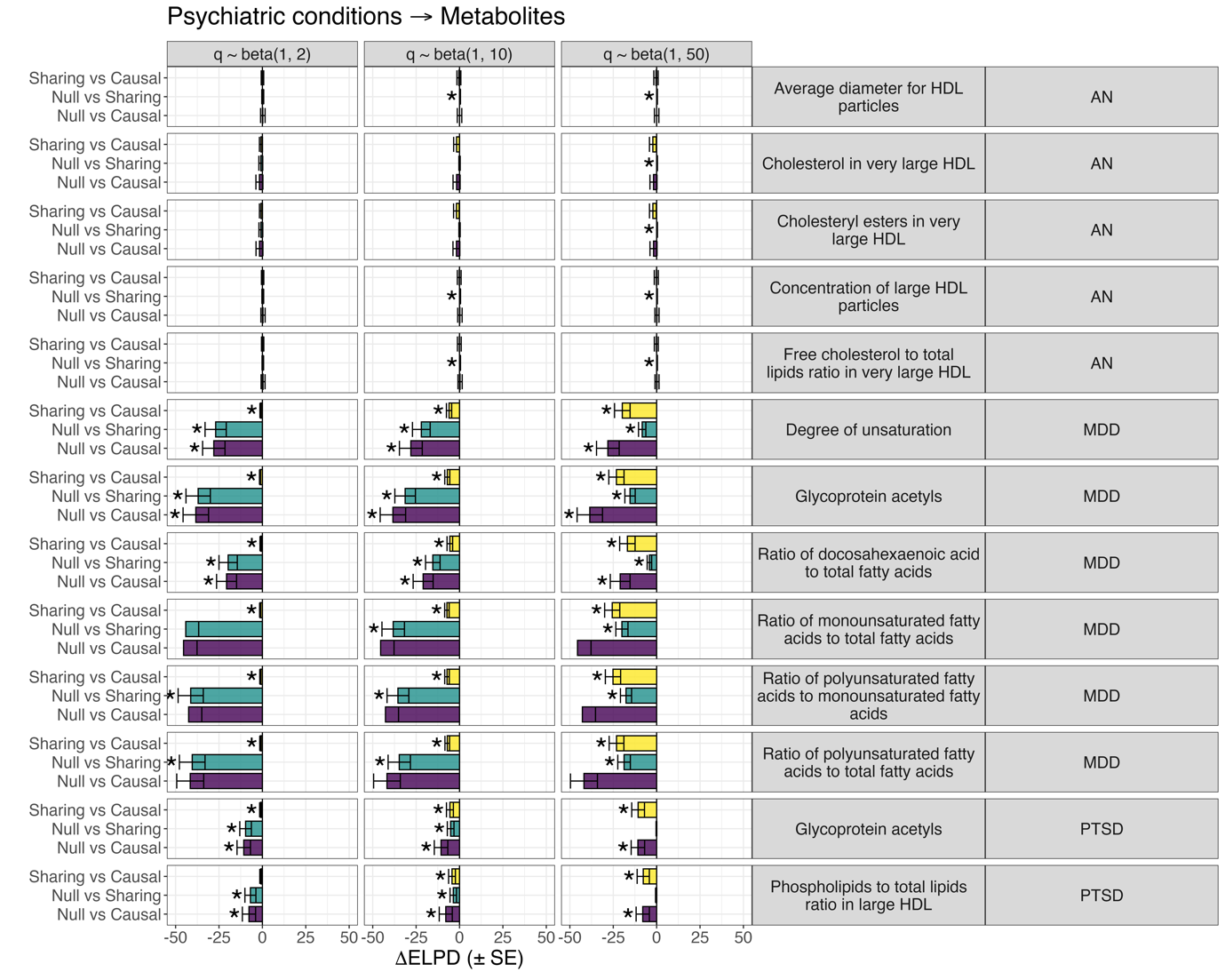
**

**Figure S2. Reverse CAUSE models examining the effect of psychiatric traits on metabolites.** *ΔELPD* (± standard error [*SE*]) estimates for CAUSE models examining the effect of psychiatric traits on metabolites, subset to trait pairings with evidence for causality in the forwards analysis. Yellow bars = the sharing versus causal model comparisons; teal bars = comparison of causal and null models; purple bars = comparison of sharing and null models. Note that a negative *ΔELPD* estimate indicates that the second model (as indicated on the left y-axis) fits better than the first. * = *P* < 0.05.


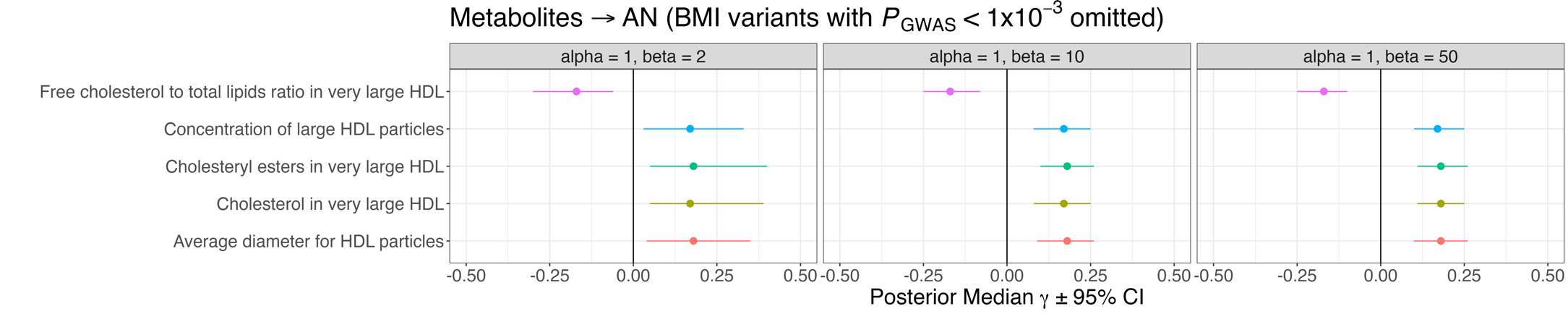


**Figure S3**. **CAUSE posterior gamma estimates for HDL-related traits on AN, with BMI-associated SNPs excluded.** Causal effect estimates (posterior median γ ± 95% credible interval [*CI*]) summarising direction of effect between all five HDL-related traits causally associated with AN, with BMI-associated variants excluded.


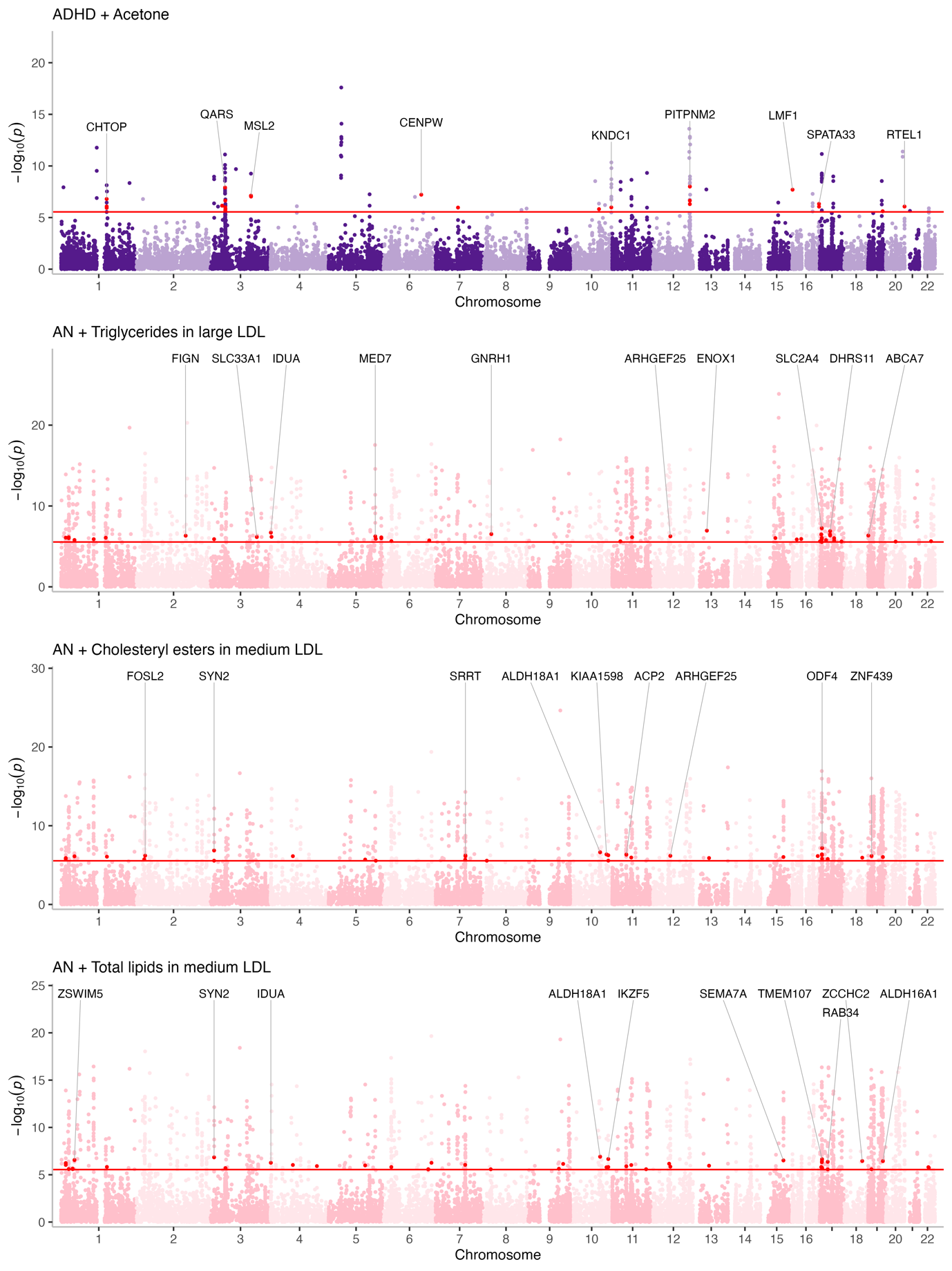


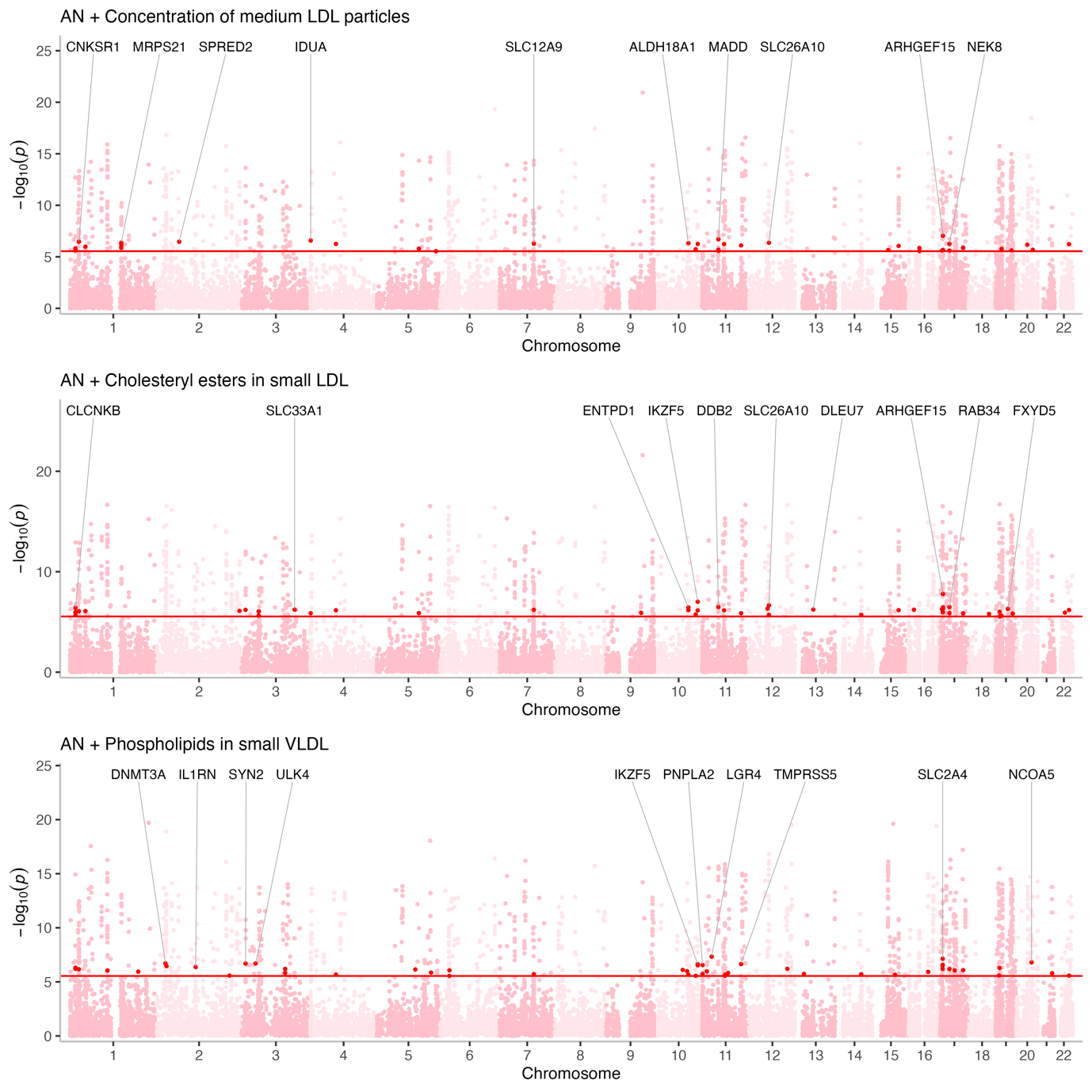


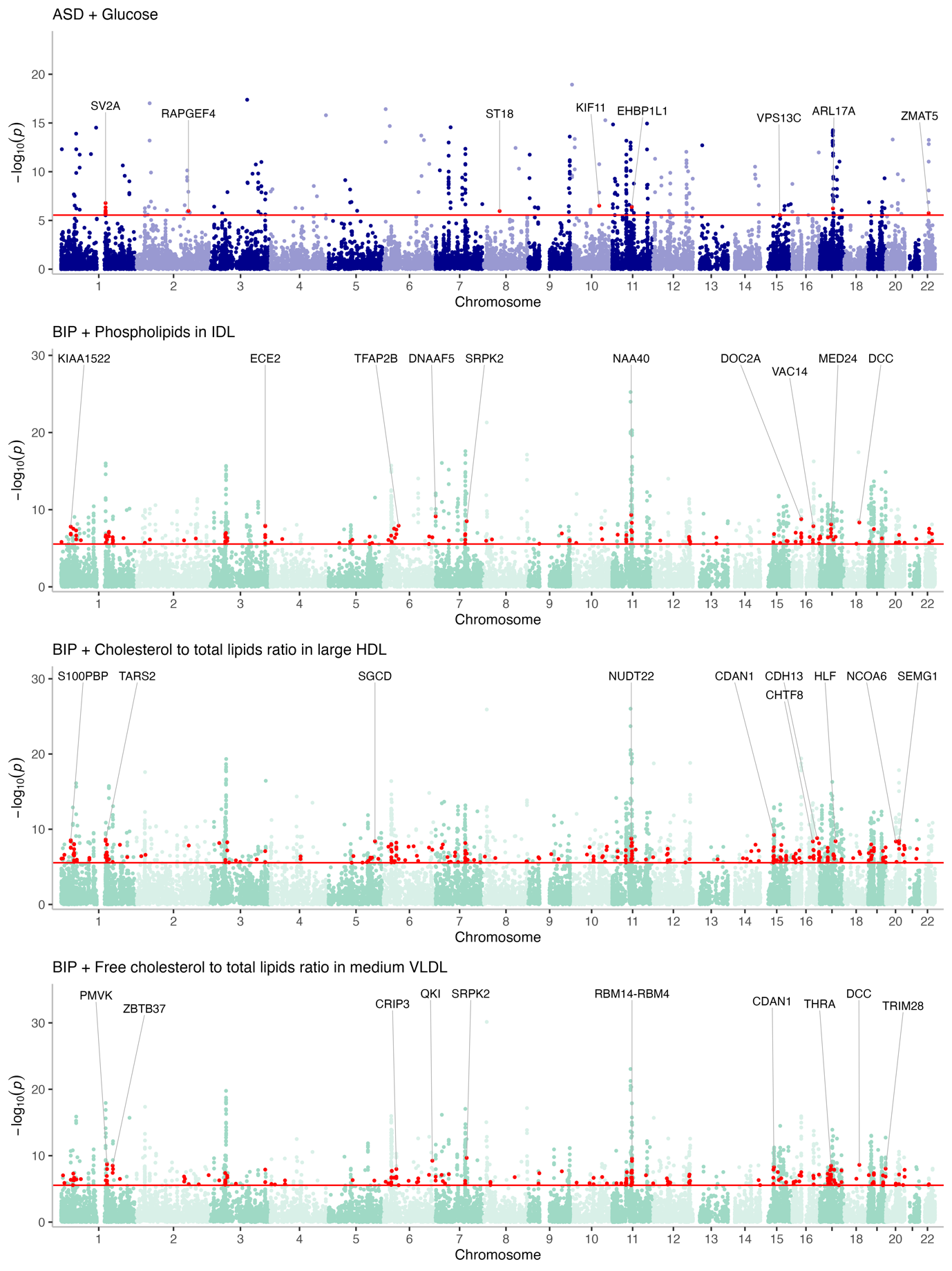


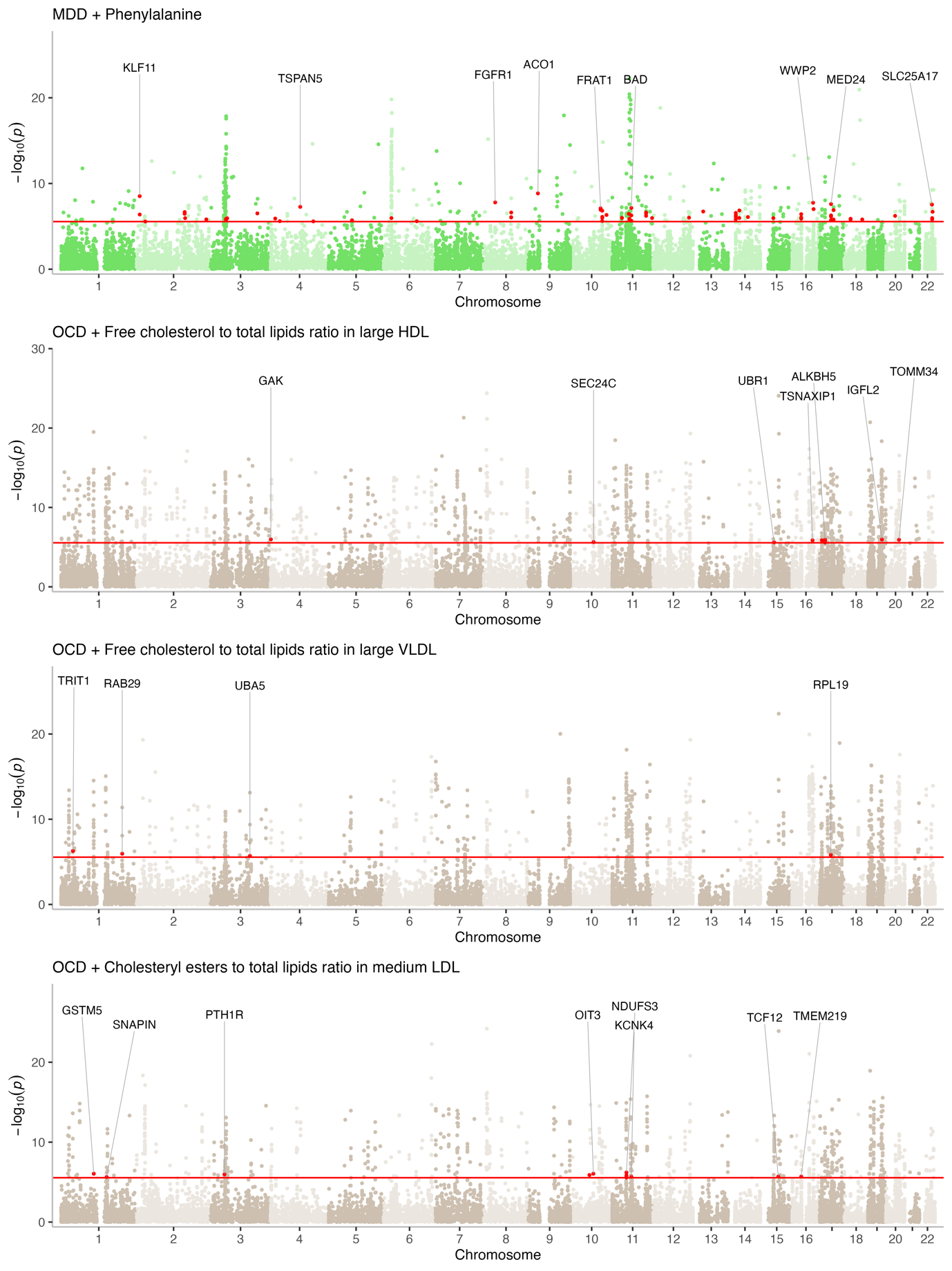


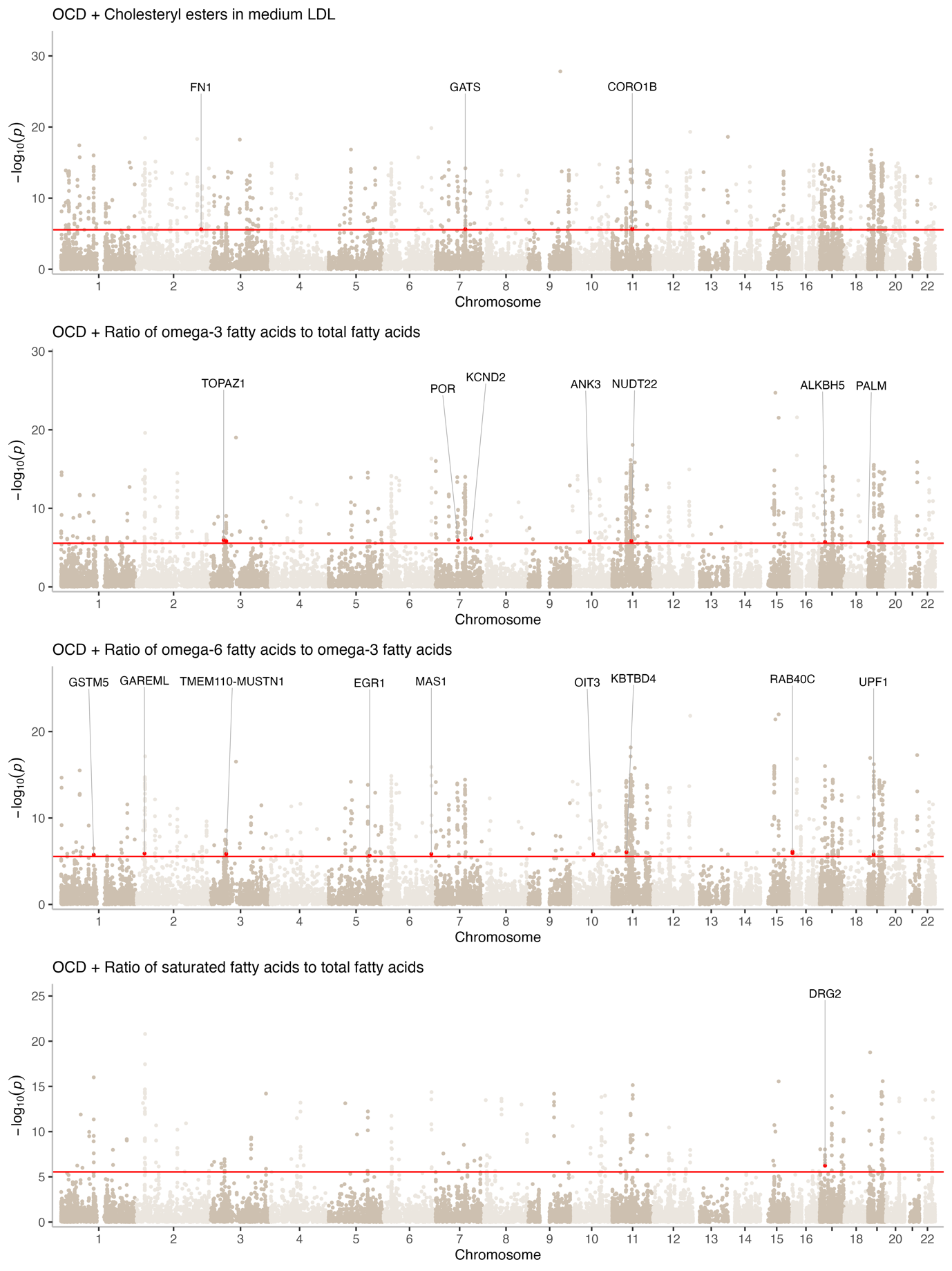


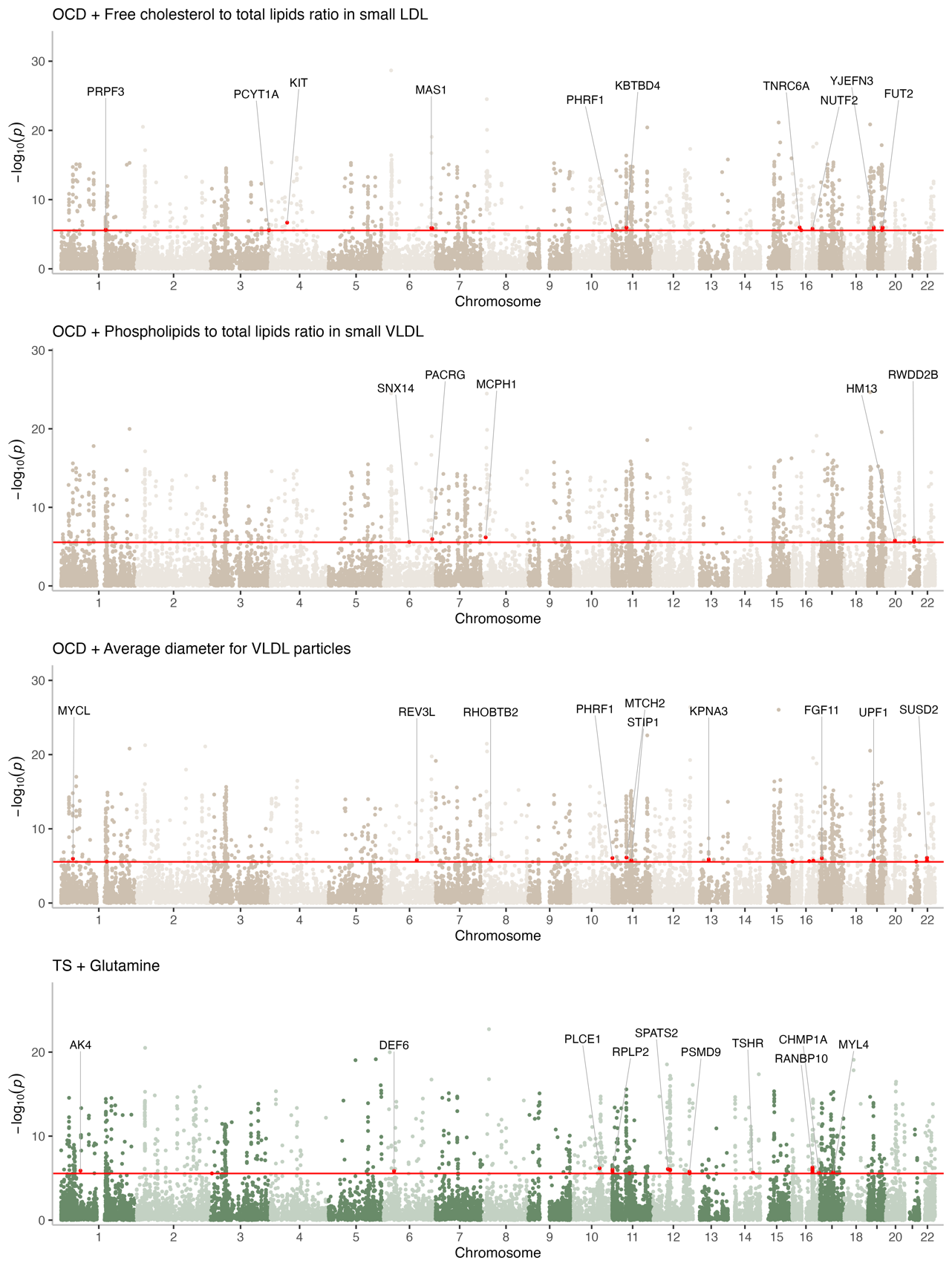


**Figure S4. Manhattan plots for all MAGMA gene-level meta-analyses.** Manhattan plots depicting pairwise MAGMA gene-level meta-analyses for all 23 metabolite-psychiatric condition pairings identified in Fig. 5a. Each point represents a single gene, with –log_10_(*P_meta_*) from the meta-analyses plotted on the y-axis. Red points = genes that were nominally significant when each trait was analysed separately but surpassed a Bonferroni correction for multiple testing (*P* < 2.6 x 10^–6^, horizontal red line) in the meta-analysis.

**REFERENCES**

1. Demontis, D., et al., *Genome-wide analyses of ADHD identify 27 risk loci, refine the genetic architecture and implicate several cognitive domains.* Nat Genet, 2023. **55**(2): p. 198-208.

2. Watson, H.J., et al., *Genome-wide association study identifies eight risk loci and implicates metabo-psychiatric origins for anorexia nervosa.* Nat Genet, 2019. **51**(8): p. 1207-1214.

3. Grove, J., et al., *Identification of common genetic risk variants for autism spectrum disorder.* Nat Genet, 2019. **51**(3): p. 431-444.

4. O'Connell, K.S., et al., *Genomics yields biological and phenotypic insights into bipolar disorder.* Nature, 2025. **639**(8056): p. 968-975.

5. Major Depressive Disorder Working Group of the Psychiatric Genomics Consortium. Electronic address, a.m.e.a.u. and C. Major Depressive Disorder Working Group of the Psychiatric Genomics, *Trans-ancestry genome-wide study of depression identifies 697 associations implicating cell types and pharmacotherapies.* Cell, 2025. **188**(3): p. 640-652 e9.

6. International Obsessive Compulsive Disorder Foundation Genetics, C. and O.C.D.C.G.A. Studies, *Revealing the complex genetic architecture of obsessive-compulsive disorder using meta-analysis.* Mol Psychiatry, 2018. **23**(5): p. 1181-1188.

7. Forstner, A.J., et al., *Genome-wide association study of panic disorder reveals genetic overlap with neuroticism and depression.* Mol Psychiatry, 2021. **26**(8): p. 4179-4190.

8. Nievergelt, C.M., et al., *Genome-wide association analyses identify 95 risk loci and provide insights into the neurobiology of post-traumatic stress disorder.* Nat Genet, 2024. **56**(5): p. 792-808.

9. Trubetskoy, V., et al., *Mapping genomic loci implicates genes and synaptic biology in schizophrenia.* Nature, 2022. **604**(7906): p. 502-508.

10. Yu, D., et al., *Interrogating the Genetic Determinants of Tourette's Syndrome and Other Tic Disorders Through Genome-Wide Association Studies.* Am J Psychiatry, 2019. **176**(3): p. 217-227.

11. Tambets, R., et al., *Genome-wide association study for circulating metabolites in 619,372 individuals.* medRxiv, 2024: p. 2024.10.15.24315557.

12. Karjalainen, M.K., et al., *Genome-wide characterization of circulating metabolic biomarkers.* Nature, 2024. **628**(8006): p. 130-138.

13. Grasby, K.L., et al., *The genetic architecture of the human cerebral cortex.* Science, 2020. **367**(6484).

14. Bulik-Sullivan, B.K., et al., *LD Score regression distinguishes confounding from polygenicity in genome-wide association studies.* Nat Genet, 2015. **47**(3): p. 291-5.

15. Bulik-Sullivan, B., et al., *An atlas of genetic correlations across human diseases and traits.* Nat Genet, 2015. **47**(11): p. 1236-41.

16. O'Connor, L.J. and A.L. Price, *Author Correction: Distinguishing genetic correlation from causation across 52 diseases and complex traits.* Nat Genet, 2018. **50**(12): p. 1753.

17. O'Connor, L.J. and A.L. Price, *Distinguishing genetic correlation from causation across 52 diseases and complex traits.* Nat Genet, 2018. **50**(12): p. 1728-1734.

18. Morrison, J., et al., *Publisher Correction: Mendelian randomization accounting for correlated and uncorrelated pleiotropic effects using genome-wide summary statistics.* Nat Genet, 2020. **52**(7): p. 750.

19. Grotzinger, A.D., et al., *Genomic structural equation modelling provides insights into the multivariate genetic architecture of complex traits.* Nat Hum Behav, 2019. **3**(5): p. 513-525.

20. Wray, N.R., et al., *Genome-wide association analyses identify 44 risk variants and refine the genetic architecture of major depression.* Nat Genet, 2018. **50**(5): p. 668-681.

21. Yehuda, R., et al., *Post-traumatic stress disorder.* Nat Rev Dis Primers, 2015. **1**: p. 15057.

22. de Leeuw, C.A., et al., *MAGMA: generalized gene-set analysis of GWAS data.* PLoS Comput Biol, 2015. **11**(4): p. e1004219.
